# Genome-wide meta-analysis in Japanese populations identifies novel variants at the *TMC6*–*TMC8* and *SIX3*–*SIX2* loci associated with HbA_1c_

**DOI:** 10.1038/s41598-017-16493-0

**Published:** 2017-11-23

**Authors:** Tsuyoshi Hachiya, Shohei Komaki, Yutaka Hasegawa, Hideki Ohmomo, Kozo Tanno, Atsushi Hozawa, Gen Tamiya, Masayuki Yamamoto, Kuniaki Ogasawara, Motoyuki Nakamura, Jiro Hitomi, Yasushi Ishigaki, Makoto Sasaki, Atsushi Shimizu

**Affiliations:** 1 Division of Biomedical Information Analysis, Iwate Tohoku Medical Megabank Organization, Disaster Reconstruction Center, Iwate Medical University, 2-1-1 Nishitokuta, Yahaba, Shiwa, Iwate, 028-3694 Japan; 2 Division of Diabetes and Metabolism, Department of Internal Medicine, School of Medicine, Iwate Medical University, 19-1 Uchimaru, Morioka, Iwate, 020-8505 Japan; 3 Division of Clinical Research and Epidemiology, Iwate Tohoku Medical Megabank Organization, Disaster Reconstruction Center, Iwate Medical University, 2-1-1 Nishitokuta, Yahaba, Shiwa, Iwate, 028-3694 Japan; 4 Department of Hygiene and Preventive Medicine, School of Medicine, Iwate Medical University, 19-1 Uchimaru, Morioka, Iwate, 020-8505 Japan; 5 Preventive Medicine and Epidemiology, Tohoku Medical Megabank Organization, Tohoku University, 2-1 Seiryo, Aoba, Sendai, 980-8573 Japan; 6 Integrative Genomics, Tohoku Medical Megabank Organization, Tohoku University, 2-1 Seiryo, Aoba, Sendai, 980-8573 Japan; 7 Iwate Tohoku Medical Megabank Organization, Disaster Reconstruction Center, Iwate Medical University, 2-1-1 Nishitokuta, Yahaba, Shiwa, Iwate, 028-3694 Japan; 8 Department of Neurosurgery, School of Medicine, Iwate Medical University, 19-1 Uchimaru, Morioka, Iwate, 020-8505 Japan; 9 Department of Internal Medicine, School of Medicine, Iwate Medical University, 19-1 Uchimaru, Morioka, Iwate, 020-8505 Japan; 10 Department of Anatomy, School of Medicine, Iwate Medical University, 2-1-1 Nishitokuda, Yahaba, Shiwa, Iwate, 028-3694 Japan; 11 Division of Innovation and Education, Iwate Tohoku Medical Megabank Organization, Disaster Reconstruction Center, Iwate Medical University, 2-1-1 Nishitokuta, Yahaba, Shiwa, Iwate, 028-3694 Japan; 12 Division of Ultrahigh Field MRI, Institute for Biomedical Sciences, Iwate Medical University, 2-1-1 Nishitokuta, Yahaba, Shiwa, Iwate, 028-3694 Japan

## Abstract

Glycated haemoglobin (HbA_1c_) is widely used as a biomarker for the diagnosis of diabetes, for population-level screening, and for monitoring the glycaemic status during medical treatment. Although the heritability of HbA_1c_ has been estimated at ~55–75%, a much smaller proportion of phenotypic variance is explained by the HbA_1c_-associated variants identified so far. To search for novel loci influencing the HbA_1c_ levels, we conducted a genome-wide meta-analysis of 2 non-diabetic Japanese populations (*n* = 7,704 subjects in total). We identified 2 novel loci that achieved genome-wide significance: *TMC6*–*TMC8* (*P* = 5.3 × 10^−20^) and *SIX3–SIX2* (*P* = 8.6 × 10^−9^). Data from the largest-scale European GWAS conducted for HbA_1c_ supported an association between the novel *TMC6–TMC8* locus and HbA_1c_ (*P* = 2.7 × 10^−3^). The association analysis with glycated albumin and glycation gap conducted using our Japanese population indicated that the *TMC6–TMC8* and *SIX3–SIX2* loci may influence the HbA_1c_ level through non-glycaemic and glycaemic pathways, respectively. In addition, the pathway-based analysis suggested that the linoleic acid metabolic and 14-3-3-mediated signalling pathways were associated with HbA_1c_. These findings provide novel insights into the molecular mechanisms that modulate the HbA_1c_ level in non-diabetic subjects.

## Introduction

The glycated haemoglobin (HbA_1c_) level represents the percentage of haemoglobin proteins bound by glucose. The glycation of haemoglobin is a non-enzymatic and predominantly irreversible reaction; therefore, the HbA_1c_ level reflects the average blood glucose level over approximately 3 months prior to the measurement^1^. Measuring HbA_1c_ is more convenient than measuring the fasting plasma glucose (FPG) level because HbA_1c_ does not need to be measured in the fasting state, has greater pre-analytical stability and is not subject to intra-individual day-to-day variability^2^. Moreover, the HbA_1c_ level, which represents long-term hyperglycaemia, is an independent risk factor for cardiovascular events^3^. Thus, HbA_1c_ is widely used as a biomarker for diagnosing diabetes, for population-level screening, and for monitoring the glycaemic status during medical treatment^4^.

Data from twin and familial studies have shown that the HbA_1c_ level is a heritable trait, with a heritability of approximately 55% to 75%^5–7^. Genome-wide association studies (GWASs) have revealed ~20 HbA_1c_-associated genetic loci^8–15^. Previous GWASs have indicated that genetic effects on the HbA_1c_ level may involve both glycaemic and non-glycaemic pathways^10,14^. HbA_1c_-associated variants located at the *ANK1*, *CDKAL1*, *G6PC2*/*ABCB11*, *GCK*, *MTNR1B*, *SLC30A8*, and *TCF7L2* loci confer an increased risk for type 2 diabetes (T2D) and/or are associated with 1 or more glycaemic traits, including FPG, 2-hour glucose, and fasting proinsulin^16^. In addition, non-glycaemic variants have been identified that are associated with HbA_1c_ but not with glycaemic traits and the T2D risk. Of these non-glycaemic variants, those at the *HFE* and *TMPRSS6* loci have been associated with red blood cell parameters^17^.

The largest-scale GWAS conducted for HbA_1c_ to date was a meta-analysis of non-diabetic European-ancestry subjects (*n* = ~46,000 subjects)^10^. The second-largest GWAS was a meta-analysis of non-diabetic East Asian populations (*n* = ~21,000 subjects)^14^. Arab, Malay and South Asian populations have also been analysed^12,15^. However, the HbA_1c_-associated variants identified to date explain a much smaller proportion of the phenotypic variance than the heritability estimates from twin and familial studies^10,14^. Accordingly, the genetic factors that influence the HbA_1c_ level have not been fully determined. To search for novel HbA_1c_-associated loci and to elucidate the molecular pathways involved in HbA_1c_ biology, we conducted a genome-wide meta-analysis of HbA_1c_ in 2 Japanese populations of non-diabetic subjects (*n* = 7,704 subjects).

## Methods

### Study subjects

Over 80,000 apparently healthy adults living in the Iwate and Miyagi Prefectures (residing along the Pacific coast of the Tohoku region of Japan) were recruited from May 2013 to March 2016 for the Tohoku Medical Megabank (TMM) Project. The study design and recruitment methods were previously described^18^. Briefly, the participants were aged from 20 to 75 years and completed questionnaires covering a wide range of topics, including sociodemographic factors, lifestyle habits, and medical history. Blood and urine tests were conducted at the baseline survey. In addition, blood samples were stored at our biobank. Participants living in the Iwate and Miyagi Prefectures were recruited by Iwate Medical University and Tohoku University, respectively. We obtained approval from the relevant ethics committees at both facilities. All participants gave written, informed consent at the time of study enrolment. This study was conducted according to the principles expressed in the Declaration of Helsinki.

The HbA_1c_ levels were measured using National Glycohemoglobin Standardization Program (NGSP)-certified methods. A high-performance liquid chromatography (HPLC) method was used for the Iwate subjects, and a latex agglutination method was used for the Miyagi subjects. We excluded diabetic participants defined based on self-reported diabetes, self-reported diabetes treatment, or HbA_1c_ ≥ 6.5%. For the Iwate subjects, FPG was measured using a hexokinase method, and glycated albumin (GA) was assayed with an enzymatic method. Glycation gaps (GGs) were calculated as the difference between the measured and GA-based predicted HbA_1c_ levels as previously described^19,20^. The plasma creatinine and cystatin C levels were measured using enzymatic and latex-coagulating nephelometry methods, respectively. The creatinine- and cystatin C-based estimated glomerular filtration rates (eGFRcrea and eGFRcys, respectively) were estimated using the Japanese equation for the eGFR calculation^21,22^. The red blood cell (RBC) counts, haemoglobin (Hb) concentrations, and haematocrit (HCT) values were measured with flow cytometry, sodium lauryl sulphate, and sheath flow detection methods, respectively. The mean corpuscular volume (MCV), mean corpuscular haemoglobin (MCH), and mean corpuscular haemoglobin concentration (MCHC) values were calculated from the RBC, Hb, and HCT values.

### Genotyping, quality control, and genotype imputation

A total of 9,966 participants enrolled in 2013 were genotyped using the HumanOmniExpressExome BeadChip Array (Illumina Inc., San Diego, CA, USA). Of these participants, 8,678 were non-diabetic with body mass index (BMI) data available. Sex was inferred using the PLINK software (version 1.90b3.45)^23,24^. Subjects in whom the inferred sex was ambiguous (*n* = 77) or inconsistent with the sex recorded in the questionnaire (*n* = 29) were excluded. In addition, subjects with a low call rate (<0.99; *n* = 8) and an estimated non-Japanese ancestry (*n* = 13; Supplementary Fig. 1) were excluded. Ancestry was estimated based on principal component analysis^25,26^. We found 1,173 close relationship pairs using the identity-by-descent method implemented in the PLINK software (PI_HAT >0.1875). We randomly excluded one of the closely related subjects for each pair; thus, 847 subjects were excluded. Single-nucleotide polymorphisms (SNPs) with low call rates (<0.95), low Hardy–Weinberg equilibrium exact test *P*-values (<1 × 10^−6^) or low minor allele frequencies (MAFs; <0.01) were filtered out. These quality-control filters resulted in the inclusion of 3,664 Iwate and 4,040 Miyagi subjects and 596,877 autosomal SNPs.

Genotype imputation was performed using the SHAPEIT (version 2.r790)^27^ and Minimac3 (version 1.0.11)^28^ software packages with the 1000 Genomes reference panel (phase 3)^29,30^. After genotype imputation, variants with a low imputation quality (*R* ^2^ < 0.8) and a low MAF (<0.01) were excluded, and 7,135,436 variants were retained for further analysis.

### Estimation of variance explained by common variants

The variance in HbA_1c_ explained by common variants was estimated based on a linear mixed model^31^ (LMM) implemented in the GCTA software (version 1.24.2)^32^. For the estimation, we additionally excluded directly genotyped SNPs with moderately low Hardy–Weinberg equilibrium exact test *P*-values (*P* < 0.05). A genetic relationship matrix (GRM) was calculated from the remaining 534,808 SNPs. Then, narrow-sense heritability was estimated with adjustments for age, sex, BMI, and recruitment site. We combined the Iwate and Miyagi subjects for this analysis.

### Association with HbA_1c_

The association between each variant and the HbA_1c_ level was tested using an LMM association method implemented in the GCTA software^32^. We modified the software to accept genotype dosage data as an input for the association test^33^. For each of the Iwate and Miyagi populations, we tested all 7,135,436 imputed variants with adjustments for age, sex, and BMI. The same GRM used in the heritability estimation was used in this analysis. Based on the summary association statistics from both populations, we performed a meta-analysis of the association between all imputed variants and the HbA_1c_ level using a fixed-effect model and the inverse-variance weighting method with the METAL software (version 2011-03-25)^34^. Variants with an association *P*-value less than the genome-wide significance (GWS; *P* < 5 × 10^−8^) were considered HbA_1c_-associated variants. All GWS variants within 500 kb were grouped into a single locus, and we determined a lead variant for each HbA_1c_-associated locus by choosing the variant with the lowest *P*-value at that locus.

### Expression quantitative trait locus (eQTL) analysis

Whole-genome and transcriptome data from 105 Japanese subjects registered in a multi-omics database (iMETHYL) were analysed to search for significant *cis*-eQTL variant-gene pairs. Pairs of a novel lead variant and neighbouring genes within ±100 kb were tested. Adaptor trimming, mapping, quality control filtering, base calling, and gene expression profiling of the iMETHYL data were previously described^35^. Briefly, genotype calling was performed with the same filtering procedures used in the 1KJPN Japanese population reference panel, including single-nucleotide variant (SNV) filtering according to read coverage, software-derived biases, departures from the Hardy–Weinberg equilibrium, and complexities of genomic regions around variants^36^. For the gene expression profiling, fragments per kb of exon per million mapped fragments (FPKM) values were calculated and normalised across subjects using the cuffquant and cuffnorm programs in the Cufflinks (version 2.2.1) software package^37^. The eQTL association was tested using linear regression and additive genetic models, *i*.*e*., log_10_ (FPKM + 1) was used as a target variable, and the genotype data (coded as 0, 1, or 2) was used as an explanatory variable. No adjustment variable was included. *P*-values < 0.05 were considered significant.

### Pathway analysis

Based on the GWAS summary data (chromosomal position and *P*-value) for the directly genotyped SNPs, gene- and pathway-based analyses were conducted using the MAGMA software (version 1.06)^38^. Variants were mapped onto protein-coding genes based on gene annotations downloaded from the NCBI Gene database (https://www.ncbi.nlm.nih.gov/gene). Then, gene-based *P*-values were calculated by aggregating variant-based *P*-values after accounting for the linkage-disequilibrium (LD) structure. The LD information was based on the East Asian population of the 1000 Genomes Project^29,30^. Pathway-based *P*-values were calculated by aggregating the gene-based *P*-values.

### Data availability

The datasets analysed in the current study are not publicly available for ethical reasons but are available upon request after approval from the Ethical Committee of Iwate Medical University, the Ethical Committee of Tohoku University, and the Materials and Information Distribution Review Committee of the TMM Project.

## Results

### Genome-wide meta-analysis in Japanese populations

**Figure 1:**
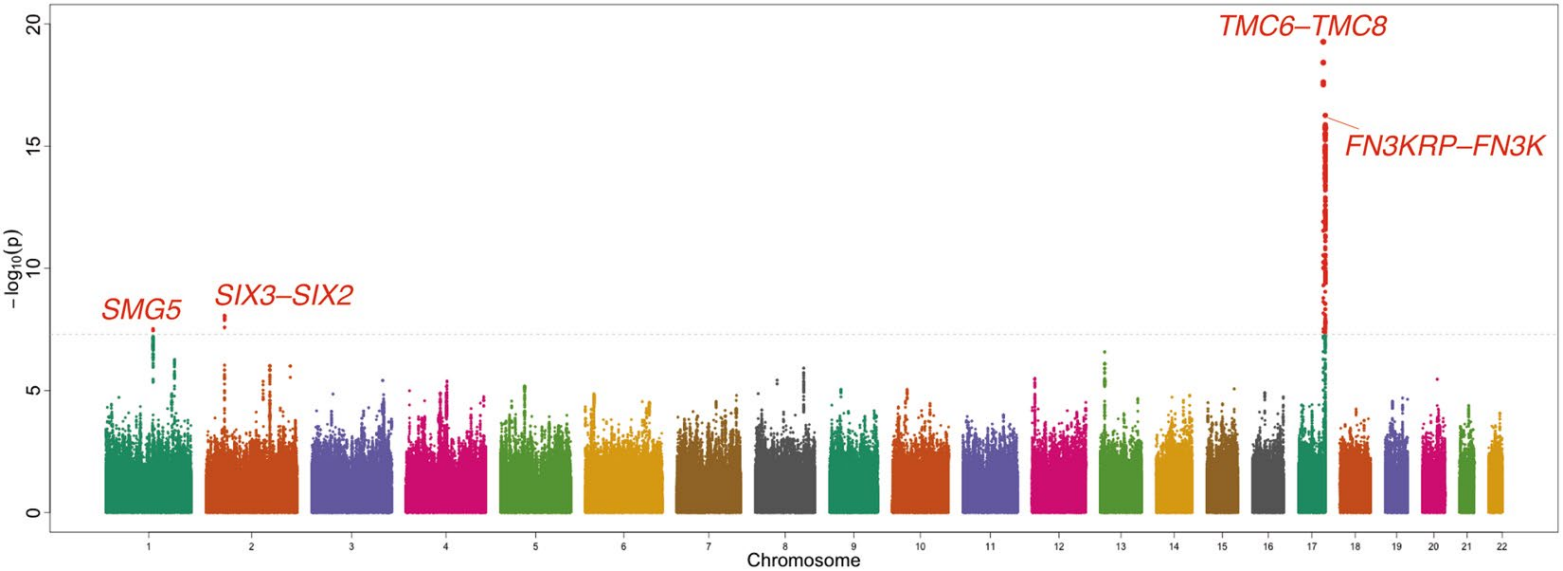
Genome-wide meta-analysis of Japanese populations. The *x*-axis represents chromosomal positions and the *y*-axis represents −log_10_ *P*-values. The grey dotted horizontal lines indicate the GWS level (*P* = 5 × 10^−8^). GWS variants were shown in red, whereas colours for other variants indicate chromosomes.

**Figure 2:**
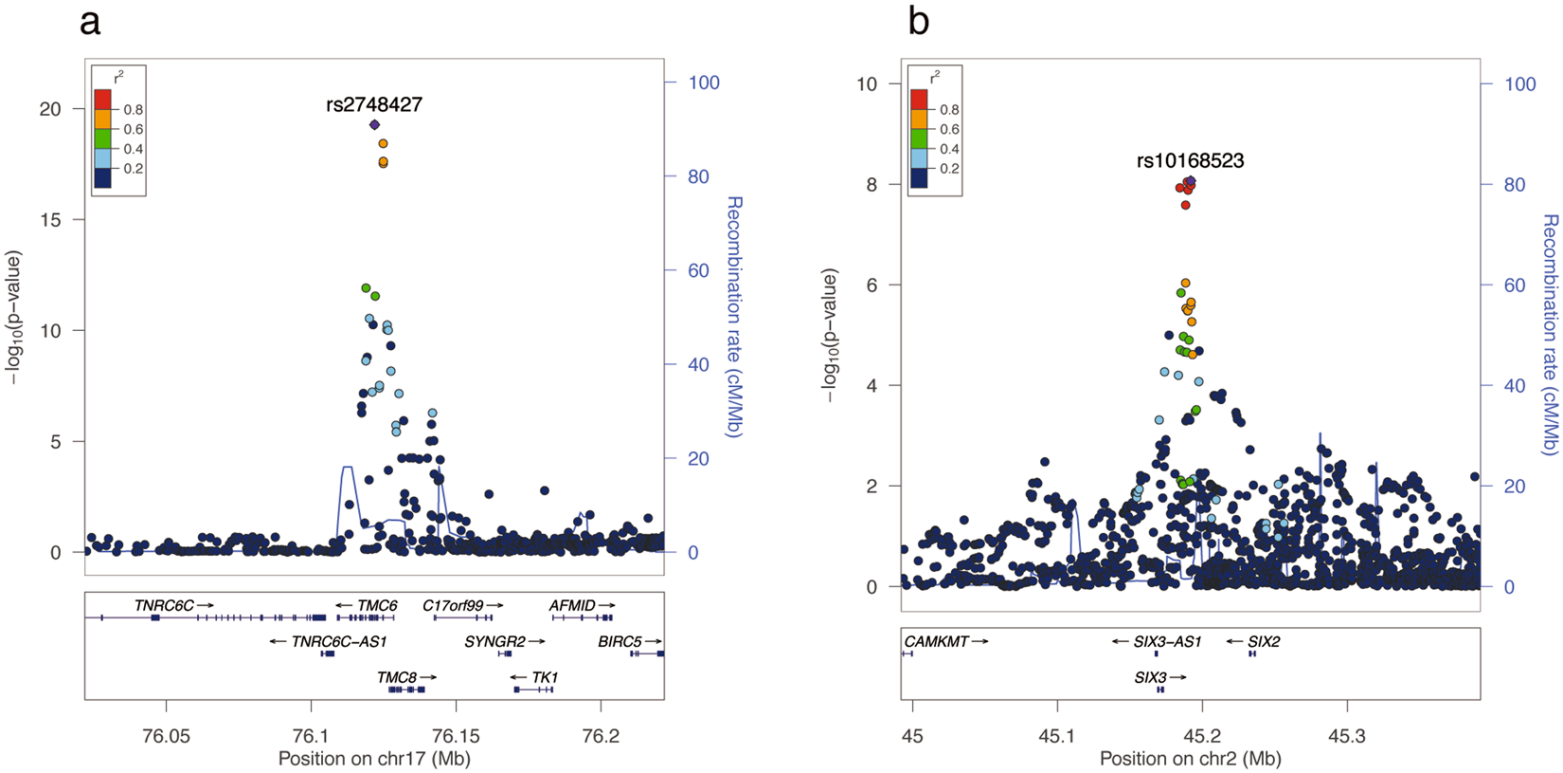
Association signals around novel lead variants. The *x*-axis represents chromosomal positions and the *y*-axis represents −log_10_ *P*-values. The lead variant is shown in purple. Colours represent the degree of LD (*r* ^2^) between each variant and the lead variant. The LD (*r* ^2^) was calculated based on the combined dataset of Iwate and Miyagi subjects. (**a**) The *TMC6–TMC8* locus. Lead variant was rs2748427. (**b**) The *SIX3–SIX2* locus. Lead variant was rs10168523.

The demographic characteristics of the study subjects are shown in Table 1 and Supplementary Table S1. In the combined Iwate and Miyagi subjects, the variance explained by common variants (MAF ≥ 0.01) was estimated to be 32.1% (standard error [SE] = 4.1%). Genome-wide association tests were conducted for each of the Iwate (*n* = 3,664) and Miyagi (*n* = 4,040) populations, and a meta-analysis was performed for the association evidence obtained from the 2 populations. The inflation factor (λ) was 1.008 (95% confidence interval [CI]: 1.006–1.009) for the Iwate population, 1.002 (95% CI: 1.000–1.003) for the Miyagi population, and 1.023 (95% CI: 1.021–1.025; Supplementary Fig. S2) for the meta-analysis, indicating that the population stratification was well-controlled. The meta-analysis showed that 4 independent loci achieved GWS (*P* < 5 × 10^−8^), as shown in Fig. 1 and Table 2. Of the 4 loci, the *TMC6*–*TMC8* locus (lead variant: rs2748427; Fig. 2a) on chromosome 17 and the *SIX3*–*SIX2* locus (lead variant: rs10168523; Fig. 2b) on chromosome 2 have not been reported by previous GWASs for HbA_1c_; therefore, these 2 loci were novel findings. The remaining 2 loci (*FN3KRP*–*FN3K* and *SMG5*) were previously reported^10,14^. No heterogeneity on the effect of the *SIX3–SIX2* lead variant rs10168523 was observed (*I* ^2^ = 0), but a large heterogeneous effect was observed for the *TMC6–TMC8* lead variant rs2748427 (*I* ^2^ = 96.8). The conditional analyses did not find additional independent signals that achieved GWS for the 2 novel loci (Supplementary Tables S2 and S3).

**Table 1:** Demographic characteristics of the study populations.

	Iwate	Miyagi
N	3,664	4,040
Female, %	66.1	68.1
Age, year (mean ± SD)	62.2 ± 10.1	58.2 ± 12.1
HbA_1c_, % (mean ± SD)	5.6 ± 0.3	5.3 ± 0.3
BMI, kg/m^2^ (mean ± SD)	23.3 ± 3.4	23.4 ± 3.5

SD, standard deviation; HbA_1c_, glycated haemoglobin; BMI, body mass index.

**Table 2:** HbA_1c_-associated lead variants.

SNP	Chr^b^	Position^c^	Gene	Rsq^d^	EA^d^	NEA^f^	Population	EAF^g^	Beta^h^	SE(Beta)^i^	*P*	*I* ^2^
rs144991356	1	156,245,918	*SMG5*	0.895	CT	C	Iwate	0.756	0.0456	0.0095	1.7E-06	
							Miyagi	0.751	0.0251	0.0077	1.1E-03	
							Meta-analysis	0.753	0.0332	0.0060	3.0E-08	64.1
**rs10168523**	**2**	**45**,**192**,**000**	***SIX3–SIX2***	**0**.**919**	**G**	**A**	**Iwate**	**0**.**457**	**0**.**0301**	**0**.**0079**	**1**.**4E-04**	
							**Miyagi**	**0**.**455**	**0**.**0279**	**0**.**0065**	**1**.**6E-05**	
							**Meta-analysis**	**0**.**456**	**0**.**0288**	**0**.**0050**	**8**.**6E-09**	**0**
**rs2748427** ^**a**^	**17**	**76**,**121**,**864**	***TMC6-TMC8***	**0**.**996**	**G**	**A**	**Iwate**	**0**.**173**	**0**.**0999**	**0**.**0099**	**6**.**1E-24**	
							**Miyagi**	**0**.**182**	**0**.**0290**	**0**.**0081**	**3**.**3E-04**	
							**Meta-analysis**	**0**.**179**	**0**.**0573**	**0**.**0063**	**5**.**3E-20**	**96**.**8**
rs35203608	17	80,681,860	*FN3KRP–FN3K*	0.960	C	CA	Iwate	0.443	0.0364	0.0078	3.1E-06	
							Miyagi	0.477	0.0441	0.0063	2.6E-12	
							Meta-analysis	0.463	0.0411	0.0049	5.5E-17	0

^a^Directly genotyped; ^b^Chromosome; ^c^Chromosomal position (GRCh37/hg19); ^d^Imputation quality in terms of R-square calculated by the Minimac3 software version 1.0.11; ^e^Effect allele; ^f^Non-efffect allele; ^g^Effect allele frequency; ^h^Effect size (HbA_1c_ difference per 1 effect allele); ^i^Standard error of effect size Results listed in bold are novel associations.

To investigate the association of the novel loci with HbA_1c_ in Europeans, we looked up the summary statistics data from the largest-scale HbA_1c_ GWAS^10^. For the *TMC6–TMC8* locus, although the lead variant rs2748427 was not found in the summary data, a proxy variant rs429216 was included (the LD *r* ^2^ between rs2748427 and rs429216 was 0.494 in East Asians and 0.615 in Europeans according to the 1000 Genomes Project data^30^ and the LDlink server^39^). The rs429216 reached GWS in our meta-analysis (Supplementary Table S2), and the association between rs429216 and HbA_1c_ was significant in the European population (*P* = 0.0027). The effect size was 0.0414% (SE = 0.0138%) in the European population. For the *SIX3–SIX2* locus, the proxy variant rs4953155 (the LD *r* ^2^ between rs10168523 and rs4953155 was 0.919 in East Asians and 0.913 in Europeans), which achieved GWS in our meta-analysis (Supplementary Table S3), was not significantly associated with HbA_1c_ in Europeans (*P* = 0.74).

**Figure 3:**
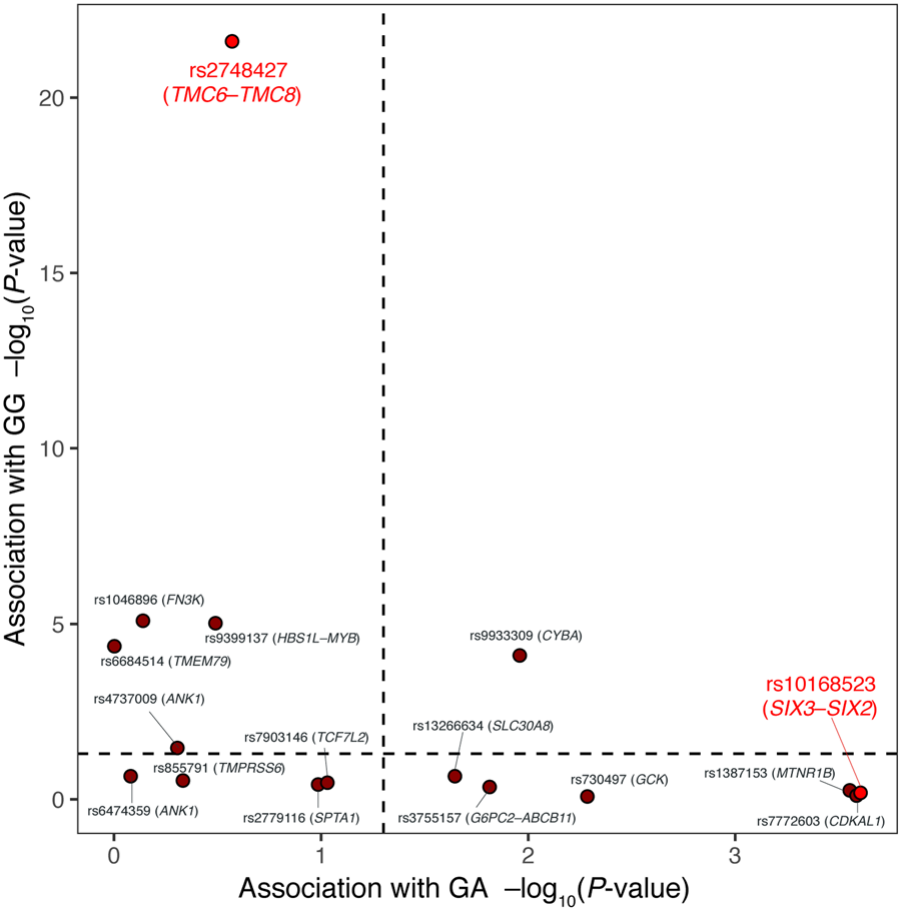
Association of genetic variants with glycated albumin and glycation gap. For novel and previously-reported HbA_1c_-associated variants, the association with glycated albumin (GA) and glycation gap (GG) was tested using the Iwate population by a linear regression model adjusted for age and sex. The *x*- and *y*-axes represent −log_10_ *P*-value of association with GA and GG, respectively. Novel lead variants were shown in red, whereas other variants were shown in brown.

To elucidate whether the 2 novel loci influenced the HbA_1c_ level through glycaemic or non-glycaemic pathways, we examined the association of the novel lead variants with GA and GG using the Iwate population. For the *TMC6–TMC8* locus, the lead variant rs2748427 was strongly associated with GG (*P* = 5.3 × 10^−23^) but not with GA (*P* = 0.65) (Fig. 3 and Supplementary Table S4), indicating that the *TMC6–TMC8* variants were non-glycaemic. For the *SIX3–SIX2* locus, the lead variant rs10168523 was associated with GA (*P* = 2.5 × 10^−4^; Fig. 3 and Supplementary Table S4) but not with GG (*P* = 0.65), suggesting that the *SIX3–SIX2* variants were glycaemic. We also examined the association of the genetic variants with the BMI, FPG, eGFRcrea, eGFRcys, and erythrocyte-related traits (RBC, Hb, HCT, MCV, MCH and MCHC) using the Iwate population. Neither novel variant was significantly associated with those variables (Supplementary Tables S4 and S5).

To examine the relationship between the novel locus and glycaemic traits, we looked up the summary statistics data available from the DIAGRAM^40,41^, MAGIC^42^, and CKDGen^43,44^ consortia. For the *TMC6–TMC8* locus, trans-ethnic T2D GWAS data^40^ from Europeans, East Asians, South Asians, Mexicans, and Mexican-Americans showed an association between the novel locus and the T2D risk (*P* = 7.0 × 10^−4^), but later European T2D GWAS data^41^ did not support this association (*P* = 0.25) (Supplementary Table S6). We did not find an appropriate proxy for rs2748427 when looked up the MAGIC data^42^ (the maximum LD *r* ^2^ in East Asians was 0.144; Supplementary Table S7). The CKDGen data^43,44^ weakly supported an association between the *TMC6–TMC8* locus and the urinary albumin-to-creatinine ratio (UACR) in nondiabetic subjects (*P* = 0.030) but did not support an association with the chronic kidney disease (CKD) risk (*P* = 0.92), eGFRcrea (*P* = 0.39), eGFRcys (*P* = 0.72), and microalbuminuria (MA; *P* = 0.81) (Supplementary Tables S8 and S9). For the *SIX3–SIX2* locus, previous GWASs of FPG showed that the locus achieved GWS in East Asian populations, although the association was not significant in Europeans^45^. Consistent with this lack of association with FPG in Europeans, variants at the *SIX3–SIX2* locus were not associated with the T2D risk, FPG, fasting insulin, beta-cell functions, CKD risk, eGFRcrea, eGFRcys, UACR, or microalbuminuria in Europeans (*P* > 0.05) (Supplementary Tables S6–S9).

### Replication analysis of previously reported variants

We examined the association of 21 previously reported lead variants^8–15^ based on our meta-analysis results. Of the lead variants, 4 were monoallelic or had a very low MAF (<0.01); therefore, these variants were excluded from this analysis. Most of the remaining 17 variants were nominally significantly associated with the HbA_1c_ level (*P* < 0.05), with the exceptions of rs7998202 (at the *ATP11A*/*TUBGCP3* locus), rs12603404 (*C17orf53*), and rs11667818 (*MYO9B*) (Table 3). The effect direction for the 14 significant variants was perfectly consistent between the Japanese and previously reported East Asian populations. Previous reports showed that the rs6474359 (*ANK1*) C allele was associated with an increased HbA_1c_ level in East Asians^14^ but with a decreased level in Europeans^10^. In the Japanese population, the C allele was associated with an increased HbA_1c_ level, which was consistent with the findings from the East Asian population.

**Table 3:** Previously reported lead variants.

SNP	Chr^b^	Position^c^	Gene(s)	Rsq^d^	EA^e^	NEA^f^	EAF^g^	Beta^h^	SE(Beta)^i^	*P*	Direction^k^
**rs6684514** ^**a**^	**1**	**156**,**255**,**456**	***TMEM79***	**1**.**000**	**G**	**A**	**0**.**782**	**0**.**0315**	**0**.**0059**	**1**.**2E-07**	**+**
**rs2779116**	**1**	**158**,**585**,**415**	***SPTA1***	**1**.**000**	**T**	**C**	**0**.**378**	**0**.**0163**	**0**.**0050**	**1**.**2E-03**	**+**
rs552976	2	169,791,438	*G6PC2/ABCB11*	0.939	A	G	0.006	NA^j^	NA^j^	NA^j^	NA
**rs3755157** ^**a**^	**2**	**169**,**792**,**171**	***G6PC2/ABCB11***	**1**.**000**	**T**	**C**	**0**.**383**	**0**.**0235**	**0**.**0051**	**4**.**4E-06**	**+**
**rs7772603**	**6**	**20**,**665**,**946**	***CDKAL1***	**0**.**989**	**C**	**T**	**0**.**411**	**0**.**0217**	**0**.**0050**	**1**.**6E-05**	**+**
rs1800562	6	26,093,141	*HFE*	0.006	A	G	0.000	NA^j^	NA^j^	NA^j^	NA
**rs9399137** ^**a**^	**6**	**135**,**419**,**018**	***HBS1L/MYB***	**1**.**000**	**T**	**C**	**0**.**652**	**0**.**0202**	**0**.**0050**	**6**.**3E-05**	**+**
**rs730497** ^**a**^	**7**	**44**,**223**,**721**	***GCK***	**1**.**000**	**A**	**G**	**0**.**175**	**0**.**0244**	**0**.**0063**	**1**.**1E-04**	**+**
**rs6474359** ^**a**^	**8**	**41**,**549**,**194**	***ANK1***	**0**.**999**	**C**	**T**	**0**.**047**	**0**.**0331**	**0**.**0115**	**4**.**1E-03**	**+**
**rs4737009** ^**a**^	**8**	**41**,**630**,**405**	***ANK1***	**0**.**996**	**A**	**G**	**0**.**436**	**0**.**0225**	**0**.**0049**	**2**.**4E-05**	**+**
**rs13266634** ^**a**^	**8**	**118**,**184**,**783**	***SLC30A8***	**0**.**994**	**C**	**T**	**0**.**583**	**0**.**0205**	**0**.**0049**	**2**.**4E-05**	**+**
rs16926246	10	71,093,392	*HK1*	0.033	T	C	0.000	NAj	NA^j^	NA^j^	NA
**rs7903146** ^**a**^	**10**	**114**,**758**,**349**	***TCF7L2***	**1**.**000**	**T**	**C**	**0**.**052**	**0**.**0279**	**0**.**0107**	**9**.**1E-03**	**+**
**rs1387153** ^**a**^	**11**	**92**,**673**,**828**	***MTNR1B***	**1**.**000**	**T**	**C**	**0**.**409**	**0**.**0143**	**0**.**0049**	**3**.**4E-03**	**+**
rs7998202^a^	13	113,331,868	*ATP11A/TUBGCP3*	0.999	A	G	0.919	0.0061	0.0087	0.48	−
rs12440118	15	42,744,094	*ZNF106*	0.121	G	A	0.002	NA^j^	NA^j^	NA^j^	NA
**rs9933309**	**16**	**88**,**844**,**932**	***CYBA***	**0**.**983**	**C**	**T**	**0**.**605**	**0**.**0145**	**0**.**0050**	**3**.**3E-03**	**+**
rs12603404^a^	17	42,223,914	*C17orf53*	1.000	G	A	0.888	0.0083	0.0076	0.27	+
**rs1046896** ^**a**^	**17**	**80**,**685**,**533**	***FN3K***	**0**.**998**	**T**	**C**	**0**.**423**	**0**.**0399**	**0**.**0048**	**1**.**7E-16**	**+**
rs11667918^a^	19	17,232,499	*MYO9B*	0.999	C	T	0.660	0.0049	0.0050	0.33	+
**rs855791** ^**a**^	**22**	**37**,**462**,**936**	***TMPRSS6***	**0**.**994**	**A**	**G**	**0**.**549**	**0**.**0102**	**0**.**0048**	**3**.**5E-02**	**+**

^a^Directly genotyped; ^b^Chromosome; ^c^Chromosomal position (GRCh37/hg19); ^d^Imputation quality in terms of R-square calculated by the Minimac3 software version 1.0.11; ^e^Effect allele; ^f^Non-efffect allele; ^g^Effect allele frequency; ^h^Effect size (HbA_1c_ difference per 1 effect allele); ^i^Standard error of effect size; ^j^Not available due to low MAF (<0.01); ^k^Effect direction in a previous GWAS in East Asians (ref.^16^) Results listed in bold were nominally significant (*P* < 0.05).

Of the 14 replicated variants in the Iwate population, 5 (rs6684514 at the *TMEM79* locus, rs9399137 at the *HBS1L/MYB* locus, rs4737009 at the *ANK1* locus, rs9933309 at the *CYBA* locus, and rs1046896 at the *FN3K* locus) were associated with GG, and 6 (rs3755157 at the *G6PC2/ABCB11* locus, rs7772603 at the *CDKAL1* locus, rs730497 at the *GCK* locus, rs13266634 at the *SLC30A8* locus, rs1387153 at the *MTNR1B* locus, and rs9933309 at the *CYBA* locus) were associated with GA (Fig. 3 and Supplementary Table S4). All 5 variants associated with GG were associated with 1 or more erythrocyte-related traits (*P* < 0.05) in the Iwate population, with the exception of rs1046896 at the *FN3K* locus (Supplementary Table S5). For the 6 variants associated with GA, 5 were associated with the T2D risk and/or FPG in the European GWAS summary data^40–42^ (Supplementary Tables S6 and S7). The lone exception was rs9933309 at the *CYBA* locus, which was also associated with GG.

### Variant functions at novel loci

For the *TMC6–TMC8* locus, we found 17 variants that met GWS (Supplementary Table S2). Most of the variants with GWS showed moderate LD with the lead variant rs2748427 (Supplementary Table S2). Two variants caused missense alterations in the TMC6 amino acid sequence (Supplementary Table S10). Based on bioinformatics analysis using SIFT^46^ and PolyPhen^47^, 1 amino acid change (rs2748427, W125R) was predicted to be a tolerated and benign variant, and 1 substitution (rs12449858, L153F) was predicted to be a deleterious and possibly damaging variant.

To interrogate the effects of the novel variants with GWS on the expression of neighbouring genes, we accessed the GTEx database^48,49^, which contains significant eQTL variant-gene pairs from 44 tissues. The alleles associated with an increased HbA_1c_ level were also associated with decreased expression levels of the *TMC6* and *TNRC6C-AS1* genes in the heart, artery, and thyroid and were associated with increased *TMC8* gene expression level in whole blood (Supplementary Table S11). Two variants were associated with increased *TMC6* gene expression level in whole blood, although 1 variant had the opposite effect.

Furthermore, we performed a *cis*-eQTL analysis using the Japanese multi-omics iMETHYL database^35^. Transcriptome data were available for purified CD4^+^ T cells and monocytes. The results showed that the lead variant rs2748427 was not associated with *TMC6* and *TMC8* gene expression in the 2 purified cell types but was associated with arylformamidase (*AFMID*) gene expression (Supplementary Table S12). The transcription start site of the *AFMID* gene is located ~62 kb from the lead variant. The rs2748427 G allele, which was associated with an increased HbA_1c_ level, was associated with decreased *AFMID* gene expression level.

For the *SIX3–SIX2* locus, 6 variants located in the intergenic regions between *SIX3* and *SIX2* achieved GWS (Supplementary Table S3). Accordingly, these 6 variants did not change the amino acid sequence of any protein-coding gene (Supplementary Table S13). No significant *cis*-eQTL was found for the *SIX3–SIX2* locus in the GTEx and iMETHYL databases (Supplementary Tables S14 and S15).

### HbA_1c_-associated molecular pathways

Variants with weak genetic effects may be clustered on certain genes even after accounting for LD, and these genes may be overrepresented in certain molecular pathways. Accordingly, we searched for molecular pathways that were collectively associated with HbA_1c_ by combining our GWAS data and prior knowledge in pathway databases. Based on the KEGG pathway database^50^, which consists of 168 pathways, the linoleic acid (LA) metabolic pathway was significantly associated after multiple testing correction (*P* < 0.05/168) (Supplementary Table S16). The pathway is composed of 34 genes, of which 12 genes were nominally associated with HbA_1c_ (*P* < 0.05). The 12 significant genes included fatty acid desaturase genes (*FADS1*, *FADS2*, and *FADS3*), cytochrome P450 enzyme genes (*CYP1A2*, *CYP2C8*, *CYP2C18*, *CYP2C19*, and *CYP2E1*), phospholipase A_2_ genes (*PLA2G2E* and *PLA2G2F*), an aldo-keto reductase gene (*AKR1B10*), and a hydroxy-delta-5-steroid dehydrogenase gene (*HSD3B7*) (Supplementary Table S17).

Based on the Ingenuity Pathway Database (http://www.ingenuity.com/index.html) analysis, the 14-3-3-mediated signalling pathway was significantly associated with HbA_1c_ (*P* < 0.05/92) (Supplementary Table S18). Of the 23 genes in the pathway, *BAD*, *CDKN1B*, *PDCD6IP*, and *VIM* were nominally associated (*P* < 0.05) (Supplementary Table S19).

We also examined the PANTHER^51^ and GO term^52^ classifications, but no pathway or gene set was associated with HbA_1c_ after Bonferroni correction (Supplementary Tables S20–S23).

Furthermore, we investigated the association of the HbA_1c_-associated KEGG LA and Ingenuity 14-3-3-mediated signalling pathways with GA, GG, FPG, eGFRcrea, eGFRcys, and erythrocyte-related traits in the Iwate population. The results showed that neither pathway was associated with any traits, with the exception of a weak association between the KEGG LA pathway and Hb (*P* = 0.045) (Supplementary Tables S24 and S25).

## Discussion

A genome-wide meta-analysis of 2 Japanese populations revealed 2 novel HbA_1c_-associated loci (*TMC6–TMC8* and *SIX3–SIX2*). The association between the *TMC6–TMC8* locus and HbA_1c_ was replicated in European populations, and the association between the *SIX3–SIX2* locus and FPG was previously reported in East Asian populations^45^. Thus, we successfully identified these 2 loci as new genetic factors influencing the HbA_1c_ levels in non-diabetic subjects. The association analysis with GA and GG indicated that the *TMC6–TMC8* locus may be involved in a non-glycaemic pathway, whereas the *SIX3–SIX2* locus may be involved in a glycaemic pathway.

GG, indicating the discordance between HbA_1c_ and other measures of glycaemic control (*e*.*g*., GA and fructosamine), has been associated with renal impairment and diabetic nephropathy^19,53–55^. GG was shown previously to be a heritable trait^56,57^. The novel HbA_1c_-associated lead variant rs2748427 at the *TMC6–TMC8* locus was strongly associated with GG in our Japanese population. This result indicated that the *TMC6–TMC8* variants may influence the HbA_1c_ level through a non-glycaemic pathway. Recent findings have indicated that the erythrocyte lifespan and glucose gradient across the erythrocyte membrane, *i*.*e*., the intracellular versus extracellular glucose concentration, may account for GG^57,58^. A previous genetic study showed an association between rs2748427 and MCV in their discovery populations with a suggestive significance (*P* = 1.6 × 10^−5^), but the association was not replicated in their replication populations^59^. In our Japanese population, the novel lead variant was not associated with any erythrocyte-related parameters, including MCV. Accordingly, the association between the *TMC6–TMC8* locus and MCV was inconclusive. Taken together, it is hypothesized that the *TMC6–TMC8* variants may affect GG through the erythrocyte life span, iron handling, glucose distribution across the erythrocyte membrane or an as-yet-undiscovered mechanism^60^.

Contrary to this hypothesis, look up of a trans-ethnic T2D GWAS showed an association between variants at the *TMC6–TMC8* locus and T2D that did not reach GWS, which could be interpreted as suggesting that the effects of the variants on HbA_1c_ may be mediated through their effects on glycaemia. However, the association was not significant in a European GWAS for T2D. In addition, we found weak genetic evidence that variants at this locus were associated with UACR in Europeans. The significance of these findings is unclear at this time.

In the eQTL analyses, we showed that variants at the *TMC6–TMC8* locus affected gene expression levels of 3 protein-coding genes, *i*.*e*., *TMC6*, *TMC8* and *AFMID*. TMC6 and TMC8 play central roles in anti-human papillomavirus (HPV) barrier. Rare loss-of-function genetic variants in either gene can lead to epidermodysplasia verruciformis (EV; OMIM 226400), which is characterized by abnormal susceptibility to specific HPVs and is associated with a high risk of skin carcinoma^61,62^. *AFMID* encodes an enzyme that converts N-formyl-L-kynurenine to L-kynurenine (KYN)^63^. In turn, KYN and several of its metabolites have an impact on insulin secretion and sensitivity^64,65^. However, it is difficult to interpret these data in the light of the associations between this locus and GG, which suggests that the effect of variants at this locus on HbA_1c_ may relate to non-glycemic determinants of HbA_1c_.

For the *SIX3−SIX2* locus, our data showing that the locus was associated with GA and evidence from a previous East Asian GWAS showing that the locus was associated with FPG consistently indicated that the locus may influence HbA_1c_ through a glycaemic pathway. Although the association between the *SIX3–SIX2* locus and FPG was not significant in our Japanese population, the association analysis may lack sufficient statistical power due to the limited number of subjects with available FPG data (*n* = 604). Previous studies showed that the effect size of the *SIX3−SIX2* variants on FPG was not heterogeneous among East Asian populations, whereas the association between the locus and FPG was not significant among European populations^45^. Our data also showed that the effect size of the locus on HbA_1c_ was not heterogeneous among 2 Japanese populations. The eQTL analysis did not identify genes with expression levels that were significantly affected by this locus. The mechanisms by which the *SIX3–SIX2* locus affects FPG and HbA_1c_ should be investigated in future studies.

A total of 7,704 non-diabetic subjects were included in our meta-analysis, making our sample smaller than the samples included in previous European^10^ (up to 46,368 subjects) and East Asian^14^ (*n* = 21,026) meta-analyses. A recent trans-ethnic genome-wide meta-analysis on HbA_1c_ identified 42 novel HbA_1c_-associated loci from an analysis of up to ~160,000 non-diabetic individuals of European, African, East Asian or South Asian ancestry^66^. The recent analysis independently found HbA_1c_-associated variants at the *TMC6*–*TMC8* locus with relatively small effect sizes (β = 0.013 [SE = 0.033; *P* = 1.3 × 10^−4^; *n* = 41,300] for Europeans, and β = 0.019 [SE = 0.059; *P* = 1.2 × 10^−3^; *n* = 9,477] for East Asians) compared to those observed in our 2 Japanese populations (Table 2). In previous meta-analyses and recent trans-ethnic studies, only one-third of the subjects were available for analysis of the *TMC6*–*TMC8* locus, possibly because less dense SNP arrays (*e*.*g*., Illumina 300 K) were included in their datasets^10,66^. A combination of effect size heterogeneity and SNP array coverage would explain why previous meta-analyses^10,14^ did not identify variants at the *TMC6*–*TMC8* locus. The recent trans-ethnic analysis did not identify variants at the *SIX3–SIX2* locus, possibly because majority of the subjects in their datasets had European ancestry, and the association between variants at the *SIX3*–*SIX2* locus and FPG/HbA_1c_ was not significant in Europeans^10,45^. The effect size heterogeneity among ethnicities would explain why the previous European meta-analysis^10^ did not detect variants at the locus.

By combining our GWAS data and pathway knowledge, we provided genetic evidence that the LA metabolic and 14-3-3-mediated signalling pathways were collectively associated with HbA_1c_. LA is abundant in vegetable oils and is a major dietary source of ω-6 polyunsaturated fatty acids (PUFAs). Arachidonic acid (AA), which is a 20-carbon ω-6 PUFA synthesized from LA, is a dominant substrate for ω-6 eicosanoids, which have pro-inflammatory activities^67,68^. Observational and interventional studies have demonstrated beneficial health outcomes of long-chain ω-3 PUFAs^69^, which competitively inhibit the synthesis of ω-6 eicosanoids from AA^67,68^. The 14-3-3 proteins integrate multiple signalling cues by recognizing post-transcriptional phosphorylation of cellular proteins and coordinating their subcellular localization^70^. The 14-3-3 proteins have been shown to protect pancreatic β-cells from pro-inflammatory cytokines by mediating pro-survival signals^71^. Knockout of the 14-3-3ζ isoform resulted in glucose intolerance and insulin resistance^72^. These data provided genetic evidence that the genetic risk for elevated HbA_1c_ levels was attributable to the LA metabolic and 14-3-3-mediated signalling pathways.

A limitation of this study is that we analysed only Japanese populations and studied GWAS summary data only from individuals with European ancestry. Although the novel *TMC6*–*TMC8* locus showed significance in both our Japanese and previous European meta-analyses, the effect size showed a large degree of heterogeneity. According to the 1000 Genomes Project data (phase 3)^29,30^, the rs2748427 G allele frequency is 0.216 in Japanese populations, which is in agreement with the value of 0.179 observed in our Japanese populations. The G allele frequency was 0.291 in East Asians, which was higher than the value observed in Europeans (0.213) and Americans (0.220) and lower than the value observed in South Asians (0.333) and Africans (0.523). The association between the novel locus and HbA_1c_ is testable in various populations, because the novel variant is common in several ethnic groups. Future studies are needed to uncover the genetic effect of the novel locus in other ethnic groups. In addition, the HbA_1c_ measurement method differed between the Iwate and Miyagi populations. HbA_1c_ levels measured using immunoassay-based methods tend to be slightly lower than those measured using HPLC-based methods^73^. Indeed, the average HbA_1c_ level in the Miyagi subjects was lower than that in the Iwate subjects (Table 1). However, we separately analysed the association between genetic variants and HbA_1c_ for each of the Iwate and Miyagi populations. Then, we performed a meta-analysis of the association evidence from the 2 Japanese populations. Accordingly, the effects of the differences in measurement methods were minimized.

In summary, we identified the *TMC6*–*TMC8* and *SIX3–SIX2* loci as novel genetic factors associated with HbA_1c_. The *TMC6–TMC8* locus may influence the HbA_1c_ level through a non-glycaemic pathway, whereas the *SIX3–SIX2* locus may affect the HbA_1c_ level via a glycaemic pathway. In addition, we provided genetic evidence that the LA metabolic and 14-3-3-mediated signalling pathways may modulate the HbA_1c_ level. Genetic evidence from this study provides insights into the molecular mechanisms that modulate the HbA_1c_ level in non-diabetic subjects.

## Electronic supplementary material

Supplementary information
