# Supplementary material for: Genome-wide meta-analysis in Japanese populations identifies novel variants at the TMC6–TMC8 and SIX3–SIX2 loci associated with HbA1c
Source: Sci Rep. 2017 Nov 23;7:16147. doi: 10.1038/s41598-017-16493-0 (PMC5701039; doi:10.1038/s41598-017-16493-0)
Supplement: Supplementary file 1 — Supplementary information [file 41598_2017_16493_MOESM1_ESM.pdf]

Supplementary information for ‘Genome-wide meta-analysis in Japanese populations identifies novel variants at the *TMC6–TMC8* and *SIX3–SIX2* loci associated with HbA_{1c}’

Tsuyoshi Hachiya, Shohei Komaki, Yutaka Hasegawa, Hideki Ohmomo, Kozo Tanno, Atsushi Hozawa, Gen Tamiya, Masayuki Yamamoto, Kuniaki Ogasawara, Motoyuki Nakamura, Jiro Hitomi, Yasushi Ishigaki, Makoto Sasaki and Atsushi Shimizu

Supplementary Figures S1–S2
Supplementary Tables S1–S25

a

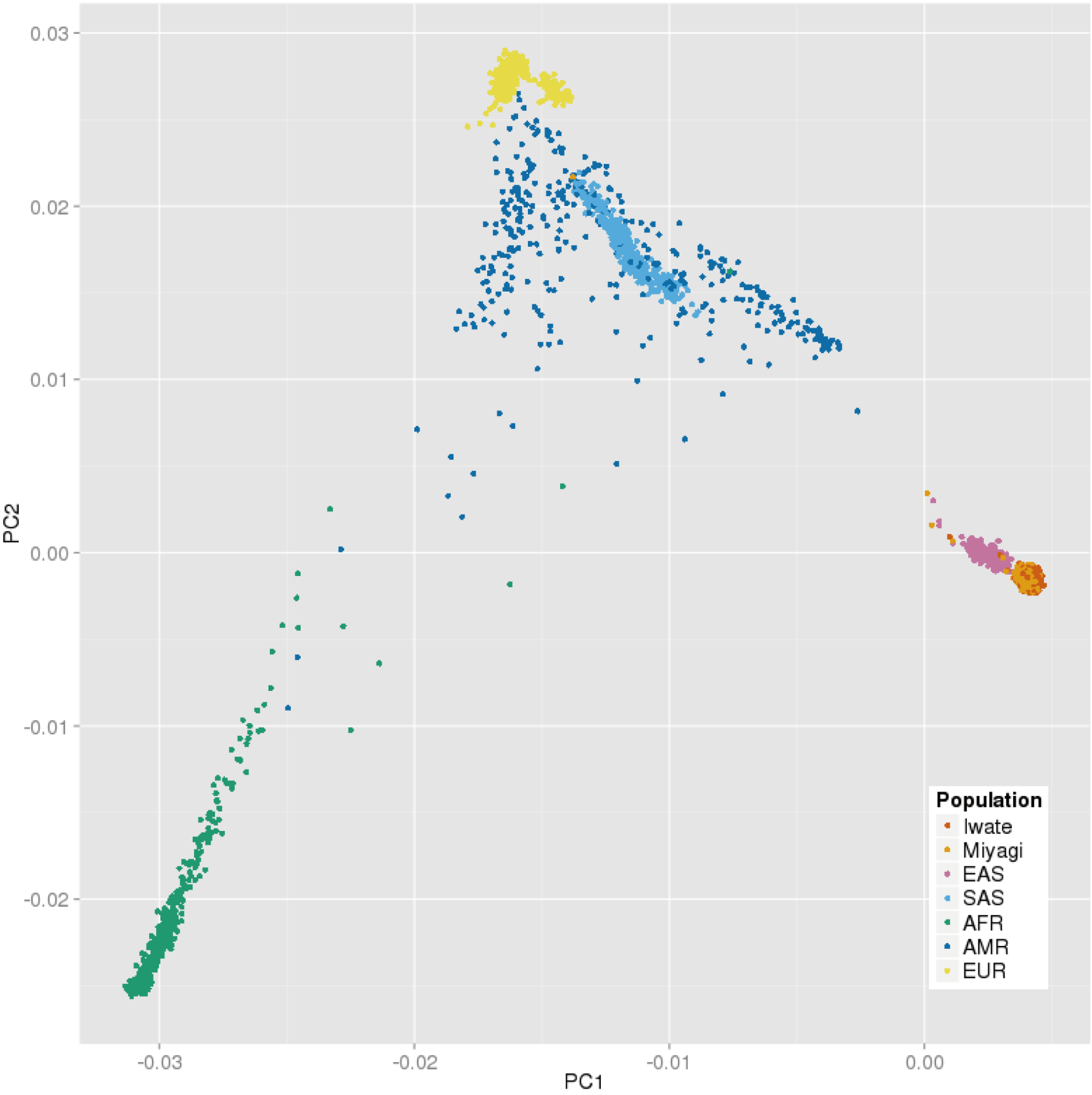

0.03
0.02
0.01
0.00
-0.01
-0.02
PC2
-0.03
-0.02
-0.01
0.00
PC1
Population
Iwate
Miyagi
EAS
SAS
AFR
AMR
EUR

b

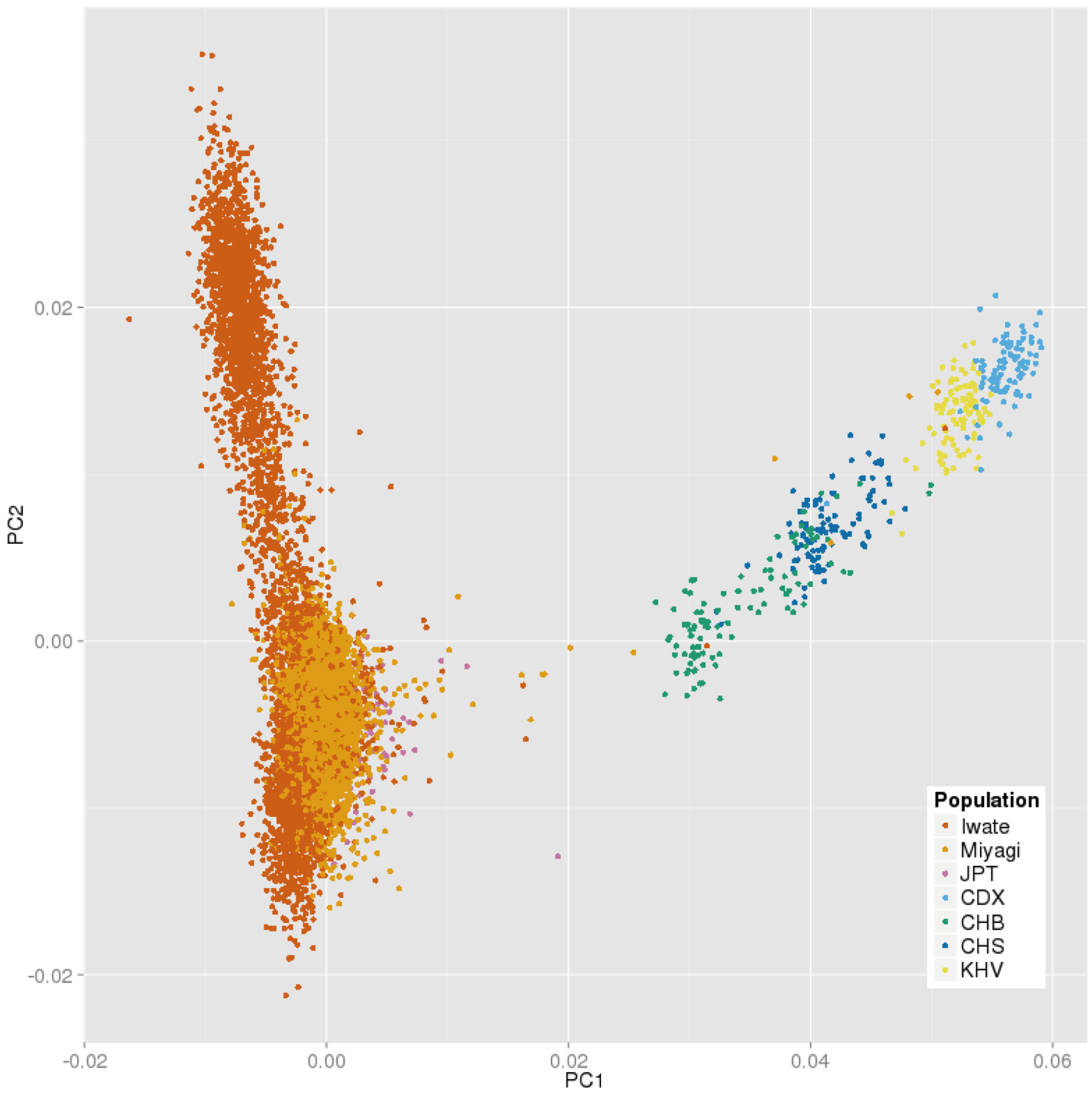

Supplementary Figure 1: Population structure of study subjects. (**a**) Principal component plot of the study subjects with 1000 Genomes Project populations. Departure from Japanese-ancestry was defined as "PC1 < 0". EAS, East Asian; SAS, South Asian; AFR, African; AMR, Ad Mixed American; EUR, European. (**b**) Principal component plot of the study subjects with 1000 Genomes Project East Asian populations. Departure from Japanese-ancestry was defined as "PC1 > 0.015". JPT, Japanese in Tokyo, Japan; CDX, Chinese Dai in Xishuangbanna, China; CHB, Han Chinese in Bejing, China; CHS, Southern Han Chinese; KHV, Kinh in Ho Chi Minh City, Vietnam.

PC, principal component

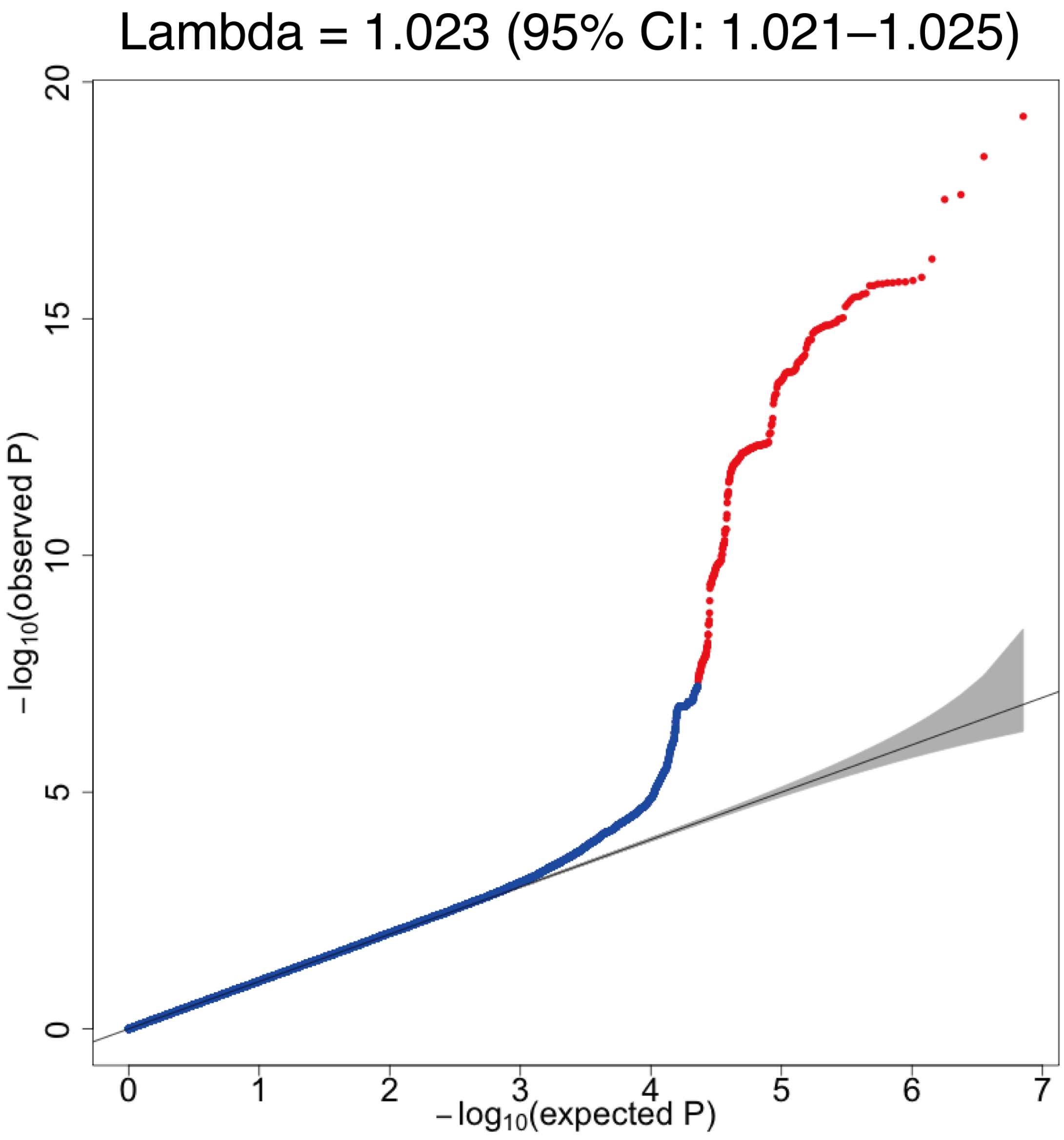

Supplementary Figure 2: A quantile-quantile plot of genome-wide meta-analysis. The *x*-axis indicates the expected -$\log_{10}$ *P*-values under the null hypothesis. The *y*-axis shows the observed -$\log_{10}$ *P*-values of association between genetic variants and HbA_{1c} level. The black line represents $y = x$, which corresponds to the null hypothesis. The gray shaded area shows 95% confidence interval of the null hypothesis. The inflation factor (lambda) is the median of the observed test statistics divided by the median of the expected test statistics. Observed *P*-values lower than genome-wide significance (GWS) were shown in red, whereas other observed *P*-values were shown in blue.

CI, confidence interval

Supplementary Table S1: Glycemic and erythrocyte-related variables of the Iwate population

Variable	Mean ± SD
FPG, mg/dL	96.7 ± 11.2
GA, %	14.5 ± 1.4
GG, %	-0.1 ± 0.4
eGFRcrea, mL/min/1.73m^2	76.6 ± 15.3
eGFRcys, mL/min/1.73m^2	100.9 ± 20.9
RBC, 10^4 cells/μL	453.3 ± 39.5
Hb, g/dL	13.6 ± 1.3
HCT, %	41.2 ± 3.5
MCV, fL	91.1 ± 4.6
MCH, pg	30.0 ± 1.8
MCHC, %	32.9 ± 0.9

FPG, fasting plasma glucose; GA, glycated albumin; GG, glycation gap; eGFRcrea, estimated glomerular filtration rate based on creatinine; cGFRcys, estimated glomerular filtration rate based on cystatin; RBC, red blood cell count; Hb, hemoglobin concentration; HCT, hematocrit value; MCV, mean corpuscular volume; MCH, mean corpuscular hemoglobin; MCHC, mean corpuscular hemoglobin concentration

Supplementary Table S2: GWS variants in the novel locus spanning *TMC6* and *TMC8* genes

SNP	Chr[b]	Position[c]	EA[d]	NEA[e]	EAF[f]	Rsq[g]	P_{GWAS}[h]	$P_{conditional}$[i]	LD r^2[j]
rs2057188	17	76,118,834	C	G	0.063	0.857	2.4E-09	5.8E-01	0.337
rs1474865	17	76,118,887	C	G	0.098	0.866	1.2E-12	6.9E-01	0.527
rs3794738	17	76,119,293	C	T	0.709	0.918	1.6E-09	1.4E-03	0.095
rs2613522	17	76,120,065	G	A	0.351	0.891	2.9E-11	1.5E-01	0.327
rs12449858[a]	17	76,121,318	G	A	0.667	0.999	5.6E-11	3.9E-04	0.107
rs2748427[a]	17	76,121,864	G	A	0.179	0.996	5.3E-20	1.000	1.000
rs761772	17	76,122,078	C	T	0.104	0.941	2.9E-12	9.6E-01	0.567
rs3813027	17	76,123,463	G	C	0.054	0.984	4.0E-08	3.9E-01	0.263
rs3813026[a]	17	76,123,528	G	A	0.053	0.999	3.0E-08	3.2E-01	0.255
rs3834968	17	76,124,810	AG	A	0.133	0.956	3.8E-19	2.3E-01	0.717
rs2748425	17	76,124,846	C	G	0.123	0.908	3.0E-18	1.9E-01	0.667
rs2748424	17	76,124,865	G	C	0.122	0.916	2.4E-18	1.8E-01	0.668
rs454139	17	76,126,094	C	T	0.074	0.979	9.3E-11	4.4E-01	0.382
rs429216[a]	17	76,126,204	G	T	0.075	0.998	5.7E-11	4.0E-01	0.382
rs379149	17	76,126,586	T	C	0.073	0.963	9.9E-11	4.3E-01	0.380
rs383603	17	76,127,431	C	G	0.404	0.962	5.0E-10	1.5E-02	0.180
rs452483	17	76,127,483	T	C	0.062	0.914	6.9E-09	5.9E-01	0.320

[a]Directly genotyped; [b]Chromosome; [c]Chromosomal position (GRCh37/hg19); [d]Effect allele; [e]Non-efffect allele; [f]Effect allele frequency; [g]Imputation quality in terms of R-square calculated by the Minimac3 software version 1.0.11; [h]*P*-values without conditioning on rs2748427; [i]*P*-values with conditioning on rs2748427; [j]Linkage disequilibrium r^2 with rs2748427

Supplementary Table S3: GWS variants in the novel locus spanning *SIX3* and *SIX2* genes

SNP[a]	Chr[b]	Position[c]	EA[d]	NEA[e]	EAF[f]	Rsq[g]	P_{GWAS}[h]	$P_{conditional}$[i]	LD r^2[j]
rs11305873	2	45,184,405	G	GT	0.451	0.964	1.2E-08	6.3E-01	0.831
rs895636	2	45,188,353	T	C	0.420	0.976	2.6E-08	9.2E-01	0.904
rs4953155	2	45,189,496	T	A	0.449	0.960	9.0E-09	9.4E-01	0.971
rs5830819	2	45,190,056	AC	A	0.424	0.969	1.3E-08	8.5E-01	0.913
rs10168523	2	45,192,000	G	A	0.456	0.919	8.6E-09	1.000	1.000
rs12712928	2	45,192,080	C	G	0.422	0.941	1.1E-08	8.2E-01	0.914

[a]All SNPs listed in this Table was not directly genotyped; [b]Chromosome; [c]Chromosomal position (GRCh37/hg19); [d]Effect allele; [e]Non-efffect allele; [f]Effect allele frequency; [g]Imputation quality in terms of R-square calculated by the Minimac3 software version 1.0.11; [h]*P*-values without conditioning on rs10168523; [i]*P*-values with conditioning on rs10168523; [j]Linkage disequilibrium r^2 with rs10168523

Supplementary Table S4: Association of novel and previously-reported loci with glycemic and kidney-related traits in the Iwate population

SNP	Gene	BMI[a]	FPG[b]	GA[a]	GG[a]	eGFRcrea[a,c]	eGFRcys[a,c]
rs10168523	*SIX3–SIX2*	2.4E-01	2.6E-01	**2.5E-04**	6.5E-01	1.1E-01	2.6E-01
rs2748427	*TMC6-TMC8*	4.7E-01	7.6E-01	2.7E-01	**2.5E-22**	1.4E-01	8.7E-01
rs6684514	*TMEM79*	9.4E-01	**2.8E-02**	1.0E+00	**4.3E-05**	8.2E-01	1.3E-01
rs2779116	*SPTA1*	2.8E-01	4.2E-01	1.0E-01	3.8E-01	8.9E-01	3.9E-01
rs3755157	*G6PC2/ABCB11*	6.7E-01	2.5E-01	**1.5E-02**	4.4E-01	8.6E-01	2.8E-01
rs7772603	*CDKAL1*	5.5E-01	2.9E-01	**2.6E-04**	7.8E-01	5.5E-01	6.1E-01
rs9399137	*HBS1L/MYB*	1.9E-01	6.6E-01	3.2E-01	**9.5E-06**	3.9E-01	7.0E-01
rs730497	*GCK*	5.8E-01	1.1E-01	**5.2E-03**	8.2E-01	7.2E-01	7.6E-02
rs6474359	*ANK1*	7.5E-01	9.8E-01	8.3E-01	2.2E-01	4.1E-01	1.1E-01
rs4737009	*ANK1*	9.2E-01	5.2E-02	5.0E-01	**3.4E-02**	6.5E-01	7.7E-01
rs13266634	*SLC30A8*	9.2E-01	1.3E-01	**2.3E-02**	2.2E-01	1.3E-01	8.9E-01
rs7903146	*TCF7L2*	3.8E-01	7.7E-01	9.3E-02	3.3E-01	5.8E-01	5.9E-01
rs1387153	*MTNR1B*	1.9E-01	6.5E-01	**2.8E-04**	5.5E-01	5.6E-01	5.3E-01
rs9933309	*CYBA*	3.4E-01	9.9E-01	**1.1E-02**	**7.9E-05**	6.2E-02	2.3E-01
rs1046896	*FN3K*	6.5E-01	2.4E-01	7.2E-01	**8.1E-06**	7.3E-01	8.2E-01
rs855791	*TMPRSS6*	4.6E-01	4.2E-01	4.6E-01	2.9E-01	9.6E-01	6.5E-02

Association between genetic variants and glycemic or kidney-related traits was tested by linear regression model with the adjustment for age and sex. *P*-values were shown in this table. Results listed in bold were nominally significant ($P < 0.05$).

[a]$n = 3,664$; [b]$n = 604$; [c]log-transformed;

BMI, body-mass index; FPG, fasting plasma glucose; GA, glycated albumin; GG, glycation gap; eGFRcrea, estimated glomerular filtration rate based on creatinine; cGFRcys, estimated glomerular filtration rate based on cystatin

Supplementary Table S5: Association of novel and previously-reported loci with erythrocyte-related traits in the Iwate population

SNP	Gene	RBC[a]	Hb[a]	HCT[a]	MCV[a]	MCH[a]	MCHC[a]
rs10168523	*SIX3–SIX2*	8.3E-01	9.9E-01	8.5E-01	4.1E-01	6.1E-01	7.4E-01
rs2748427	*TMC6-TMC8*	6.5E-02	4.8E-01	3.6E-01	8.9E-02	9.0E-02	7.3E-01
rs6684514	*TMEM79*	2.7E-01	1.5E-01	8.6E-01	1.7E-01	5.3E-01	**1.2E-04**
rs2779116	*SPTA1*	3.7E-01	7.6E-01	3.3E-01	**2.7E-03**	3.1E-01	**1.4E-03**
rs3755157	*G6PC2/ABCB11*	2.7E-01	2.4E-01	1.5E-01	6.9E-01	9.6E-01	7.0E-01
rs7772603	*CDKAL1*	1.4E-01	**4.4E-02**	9.3E-02	9.6E-01	5.7E-01	1.4E-01
rs9399137	*HBS1L/MYB*	**9.6E-15**	**1.0E-02**	**4.0E-05**	**3.7E-11**	**2.6E-13**	**8.3E-04**
rs730497	*GCK*	4.5E-01	5.0E-01	2.8E-01	6.9E-01	9.0E-01	4.2E-01
rs6474359	*ANK1*	3.1E-01	9.9E-01	8.4E-01	1.9E-01	1.6E-01	5.6E-01
rs4737009	*ANK1*	**1.1E-02**	**1.8E-02**	1.0E-01	5.1E-02	6.2E-01	**2.0E-02**
rs13266634	*SLC30A8*	8.1E-01	5.3E-01	7.7E-01	9.3E-01	7.1E-01	3.2E-01
rs7903146	*TCF7L2*	4.1E-01	6.5E-02	1.8E-01	5.6E-01	1.9E-01	1.0E-01
rs1387153	*MTNR1B*	2.7E-01	5.9E-01	4.9E-01	3.9E-01	4.1E-01	7.3E-01
rs9933309	*CYBA*	9.6E-02	7.5E-01	4.9E-01	**1.2E-04**	**4.5E-02**	**1.0E-02**
rs1046896	*FN3K*	3.1E-01	2.3E-01	1.8E-01	8.3E-01	9.7E-01	8.5E-01
rs855791	*TMPRSS6*	9.5E-01	**7.1E-04**	**2.0E-02**	**4.9E-04**	**3.5E-06**	**1.4E-03**

Association between genetic variants and glycemic or kidney-related traits was tested by linear regression model with the adjustment for age and sex. *P*-values were shown in this table. Results listed in bold were nominally significant ($P < 0.05$).

[a]$n = 3,664$

RBC, red blood cell count; Hb, hemoglobin concentration; HCT, hematocrit value; MCV, mean corpuscular volume; MCH, mean corpuscular hemoglobin; MCHC, mean corpuscular hemoglobin concentration

Supplementary Table S6: Association of novel and previously-reported loci with risk of type 2 diabetes from look up of GWAS summary statistics available from DIAGRAM consortium

SNP	Gene	Trans-ethnic GWAS		European GWAS	
		n	*P*	*n*	*P*
rs10168523	*SIX3–SIX2*	11,322[a]	9.8E-01[a]	44,414	1.0E-01
rs2748427	*TMC6-TMC8*	11,893[b]	**7.0E-04**[b]	36,081	2.5E-01
rs6684514	*TMEM79*	104,550	4.9E-02	44,414	3.7E-01
rs2779116	*SPTA1*	90,572	7.2E-01	44,414	1.1E-01
rs3755157	*G6PC2/ABCB11*	104,448	1.9E-01	44,414	5.2E-02
rs7772603	*CDKAL1*	86,726	**5.3E-25**	44,414[c]	**6.4E-16**[c]
rs9399137	*HBS1L/MYB*	79,728	1.0E-01	44,414	8.4.E-01
rs730497	*GCK*	104,570	5.5E-02	44,414	1.6.E-01
rs6474359	*ANK1*	101,871	7.0E-01	44,414	7.4.E-02
rs4737009	*ANK1*	103,247	1.7E-01	44,414	1.0.E-01
rs13266634	*SLC30A8*	102,567	**2.7E-20**	44,414	**7.6.E-08**
rs7903146	*TCF7L2*	104,575	**7.8E-75**	44,414	**8.6.E-62**
rs1387153	*MTNR1B*	100,588	**5.7E-06**	44,414	**2.8.E-04**
rs9933309	*CYBA*	84,699	9.1E-01	36,081	4.2.E-01
rs1046896	*FN3K*	110,192	5.2E-01	44,414	5.4E-01
rs855791	*TMPRSS6*	98,005	6.8E-01	40,436	2.7E-01

Results listed in bold were nominally significant ($P < 0.05$).

[a]Proxy SNP rs4953155 (LD r2 in East Asians was 0.919) was used; [b]Proxy SNP rs383603 (LD r^2 in East Asians was 0.339) was used; [c]Proxy SNP rs6456367 (LD r^2 in East Asians was 1.000) was used

Supplementary Table S7: Association of novel and previously-reported loci with glycemic traits from look up of GWAS summary statistics available from MAGIC consortium

SNP	Gene	FPG	Fasting insulin[a]	HOMA-B[a]	HOMA-IR[a]
rs10168523	*SIX3–SIX2*	2.0E-01[b]	7.0E-01[b]	9.6E-01[b]	7.4E-01[b]
rs2748427	*TMC6-TMC8*	NA[c]	NA[c]	NA[c]	NA[c]
rs6684514	*TMEM79*	**2.2E-02**	**3.0E-02**	2.0E-01	**1.1E-02**
rs2779116	*SPTA1*	7.2E-01[d]	1.0E-01[d]	6.2E-02[d]	1.1E-01[d]
rs3755157	*G6PC2/ABCB11*	**1.9E-09**	7.8E-01	**2.7E-03**	1.4E-01
rs7772603	*CDKAL1*	**1.3E-02**	1.1E-01	5.3E-02	2.2E-01
rs9399137	*HBS1L/MYB*	**1.3E-03**	9.4E-01	1.1E-01	7.5E-01
rs730497	*GCK*	**2.5E-35**	2.8E-01	**3.8E-08**	**9.9E-03**
rs6474359	*ANK1*	6.2E-01	1.0E-01	**4.8E-02**	3.1E-01
rs4737009	*ANK1*	2.7E-01	1.8E-01	5.0E-02	1.4E-01
rs13266634	*SLC30A8*	**5.5E-10**	4.4E-01	**2.4E-05**	9.7E-01
rs7903146	*TCF7L2*	**2.8E-08**	**4.6E-03**	**1.4E-07**	**3.4E-02**
rs1387153	*MTNR1B*	**6.6E-45**	4.4E-01	**1.1E-14**	2.2E-01
rs9933309	*CYBA*	6.0E-01	3.7E-01	1.9E-01	3.1E-01
rs1046896	*FN3K*	2.4E-01	3.5E-01	5.5E-01	4.9E-01
rs855791	*TMPRSS6*	9.5E-02	2.5E-01	8.2E-01	2.5E-01

Results listed in bold were nominally significant ($P < 0.05$).

[a]log-transformed; [b]Proxy SNP rs895636 (LD r^2 in East Asians was 0.742) was used; [c]Any appropriate proxy SNP could not be found (maximum LD r^2 in East Asians was 0.144 [rs3813026]); [d]Proxy SNP rs2157691 (LD r^2 in East Asians was 1.000) was used

Supplementary Table S8: Association of novel and previously-reported loci with CKD risk and eGFR from look up of GWAS summary statistics available from CKDGen consortium

SNP	Gene	CKD risk	eGFRcrea			eGFRcys
			All subjects	DM subjects	Non-DM subjects	
rs10168523	*SIX3–SIX2*	8.1E-01[a]	7.7E-01[a]	6.9E-01[a]	7.9E-01[a]	6.1E-01[a]
rs2748427	*TMC6-TMC8*	9.2E-01[b]	3.9E-01[b]	8.8E-01[b]	3.8E-01[b]	7.2E-01[b]
rs6684514	*TMEM79*	6.0E-02	6.5E-01	1.4E-01	9.9E-01	1.5E-01
rs2779116	*SPTA1*	5.4E-01[c]	5.1E-02[c]	9.3E-01[c]	5.4E-02[c]	9.5E-01[c]
rs3755157	*G6PC2/ABCB11*	4.9E-01	7.8E-01	6.7E-01	8.7E-01	9.0E-01
rs7772603	*CDKAL1*	2.9E-01	9.5E-01	3.2E-01	4.2E-01	7.7E-01
rs9399137	*HBS1L/MYB*	9.7E-01	2.5E-01	5.3E-01	1.7E-01	3.8E-01
rs730497	*GCK*	6.1E-01	7.7E-01	3.6E-01	8.6E-01	4.4E-01
rs6474359	*ANK1*	8.2E-01[d]	5.7E-02[d]	1.9E-01[d]	9.3E-02[d]	4.4E-01[d]
rs4737009	*ANK1*	1.6E-01	7.0E-01	6.1E-01	5.1E-01	3.2E-01
rs13266634	*SLC30A8*	9.6E-01	9.2E-01	4.7E-01	4.5E-01	6.6E-01
rs7903146	*TCF7L2*	4.6E-01	3.0E-01	5.0E-01	2.4E-01	9.4E-01
rs1387153	*MTNR1B*	6.8E-01	6.6E-01	8.5E-01	6.7E-01	7.4E-01
rs9933309	*CYBA*	8.5E-02	6.0E-01	3.0E-01	6.5E-01	3.1E-01
rs1046896	*FN3K*	9.6E-01	6.6E-01	5.6E-01	5.0E-01	5.0E-01
rs855791	*TMPRSS6*	7.5E-01	7.7E-01	4.1E-01	3.7E-01	8.9E-01

[a]Proxy SNP rs895636 (LD r^2 in East Asians was 0.742) was used; [b]Proxy SNP rs383603 (LD r^2 in East Asians was 0.339) was used; [c]Proxy SNP rs2157691 (LD r^2 in East Asians was 1.000) was used; [d]Proxy SNP rs6474360 (LD r^2 in East Asians was 0.750) was used

CKD, chronic kidney disease; eGFRcre, estimated glomerular filtration rate based on creatinine; eGFRcys, estimated glomerular filtration rate based on cystatin; DM, diabetes mellitus

Supplementary Table S9: Association of novel and previously-reported loci with UACR and microalbuminuria from look up of GWAS summary statistics available from CKDGen consortium

SNP	Gene	UACR			MA
		All subjects	DM subjects	Non-DM subjects	
rs10168523	*SIX3–SIX2*	4.5E-01[a]	7.7E-01[a]	2.0E-01[a]	7.9E-01[a]
rs2748427	*TMC6-TMC8*	6.6E-02[b]	3.9E-01[b]	**3.0E-02[b]**	8.1E-01[b]
rs6684514	*TMEM79*	3.2E-01	2.2E-01	4.6E-01	9.6E-01
rs2779116	*SPTA1*	7.8E-01[c]	6.4E-01[c]	6.7E-01[c]	9.7E-01[c]
rs3755157	*G6PC2/ABCB11*	6.6E-01	2.6E-01	7.5E-01	4.8E-01
rs7772603	*CDKAL1*	9.9E-01	4.3E-01	2.4E-01	1.3E-01
rs9399137	*HBS1L/MYB*	4.8E-01	9.9E-01	6.9E-01	9.7E-01
rs730497	*GCK*	5.7E-01	6.9E-01	7.6E-01	3.7E-01
rs6474359	*ANK1*	9.4E-01[d]	7.1E-01[d]	9.7E-01[d]	6.5E-01[d]
rs4737009	*ANK1*	3.2E-01	3.9E-01	2.0E-01	2.1E-01
rs13266634	*SLC30A8*	4.9E-01	**8.5E-03**	2.5E-01	5.7E-01
rs7903146	*TCF7L2*	9.7E-01	**1.1E-02**	8.0E-01	5.2E-01
rs1387153	*MTNR1B*	9.0E-01	7.9E-01	8.6E-01	1.2E-01
rs9933309	*CYBA*	9.4E-01	1.7E-01	5.0E-01	7.4E-01
rs1046896	*FN3K*	**1.7E-02**	2.4E-01	**4.6E-02**	2.1E-01
rs855791	*TMPRSS6*	1.4E-01	4.1E-01	3.2E-01	6.0E-02

[a]Proxy SNP rs895636 (LD r^2 in East Asians was 0.742) was used; [b]Proxy SNP rs383603 (LD r^2 in East Asians was 0.339) was used; [c]Proxy SNP rs2157691 (LD r^2 in East Asians was 1.000) was used; [d]Proxy SNP rs6474360 (LD r^2 in East Asians was 0.750) was used

UACR, urinary albumin-to-creatinine ratio; chronic kidney disease; MA, microalbuminuria defined as UACR > 25mg/g in women and >17 mg/g in men; DM, diabetes mellitus

Supplementary Table S10: Functional annotation for GWS variants in the novel locus spanning *TMC6* and *TMC8* genes

SNP	Gene	Transcript ID	Effect	SIFT	PolyPhen
rs2057188	*TMC6*	NM_001127198	Splice region variant	-	-
rs1474865	*TMC6*	NM_001127198	Intron variant	-	-
rs3794738	*TMC6*	NM_001127198	Intron variant	-	-
rs2613522	*TMC6*	NM_001127198	Splice region variant	-	-
rs12449858	*TMC6*	NM_001127198	Missense variant (L153F)	deleterious	possibly damaging
rs2748427	*TMC6*	NM_001127198	Missense variant (W125R)	tolerated	benign
rs761772	*TMC6*	NM_001127198	Intron variant	-	-
rs3813027	*TMC6*	NM_001127198	Intron variant	-	-
rs3813026	*TMC6*	NM_001127198	Intron variant	-	-
rs3834968	*TMC6*	NM_001127198	Upstream gene variant	-	-
rs2748425	*TMC6*	NM_001127198	Upstream gene variant	-	-
rs2748424	*TMC6*	NM_001127198	Upstream gene variant	-	-
rs454139	*TMC6*	NM_001127198	Upstream gene variant	-	-
rs429216	*TMC6*	NM_001127198	Upstream gene variant	-	-
rs379149	*TMC6*	NM_001127198	Upstream gene variant	-	-
rs383603	*TMC8*	NM_152468	5 prime UTR variant	-	-
rs452483	*TMC8*	NM_152468	5 prime UTR variant	-	-

Supplementary Table S11: eQTL results from the GTEx database for GWS variants in the novel locus spanning *TMC6* and *TMC8* genes

SNP	EA[a]	GTEx[b]
rs2057188	C	*TMC6* (Heart - Atrial Appendage [-]; Heart - Left Ventricle [-])<br>*TNRC6C-AS1* (Heart - Atrial Appendage [-]; Heart - Left Ventricle [-])<br>*TMC8* (Whole Blood [+])
rs1474865	C	*TMC6* (Heart - Atrial Appendage [-]; Heart - Left Ventricle [-]; Artery - Tibial [-])<br>*TNRC6C-AS1* (Heart - Atrial Appendage [-]; Heart - Left Ventricle [-]; Thyroid [-])<br>*TMC8* (Whole Blood [+])
rs3794738	C	No significant eQTLs were found in all eQTL Tissues
rs2613522	G	*TMC6* (Heart - Atrial Appendage [-])
rs12449858	G	No significant eQTLs were found in all eQTL Tissues
rs2748427	G	*TMC6* (Heart - Atrial Appendage [-]; Heart - Left Ventricle [-])<br>*TNRC6C-AS1* (Heart - Left Ventricle [-])
rs761772	C	*TMC6* (Heart - Atrial Appendage [-]; Heart - Left Ventricle [-]; Artery - Tibial [-])<br>*TNRC6C-AS1* (Heart - Atrial Appendage [-]; Heart - Left Ventricle [-]; Thyroid [-])<br>*TMC8* (Whole Blood [+])
rs3813027	G	*TMC6* (Whole Blood [+]; Thyroid [+])<br>*TNRC6C-AS1* (Whole Blood [+]; Thyroid [+])
rs3813026	G	*TMC6* (Whole Blood [+]; Thyroid [+])<br>*TNRC6C-AS1* (Whole Blood [+])
rs3834968	AG	Not available
rs2748425	C	*TMC6* (Heart - Atrial Appendage [-]; Heart - Left Ventricle [-])<br>*TNRC6C-AS1* (Heart - Left Ventricle [-])<br>*TMC8* (Whole Blood [+])
rs2748424	G	*TMC6* (Heart - Atrial Appendage [-]; Heart - Left Ventricle [-])<br>*TNRC6C-AS1* (Heart - Left Ventricle [-])<br>*TMC8* (Whole Blood [+])

[a]Effect allele; [b]Significant eQTL results (gene name, tissue, and effect direction) were shown. Tissues and effect directions were shown in parentheses '()' and brackets '[]', respectively.

Supplementary Table S11: eQTL results from the GTEx database for GWS variants in the novel locus spanning *TMC6* and *TMC8* genes (cont.)

SNP	EA[d]	GTEx
rs454139	C	*TMC6* (Heart - Atrial Appendage [-]; Heart - Left Ventricle [-]; Artery - Tibial [-]; Thyroid [-])<br>*TNRC6C-AS1* (Heart - Atrial Appendage [-]; Heart - Left Ventricle [-]; Thyroid [-])<br>*TMC8* (Whole Blood [+])
rs429216	G	*TMC6* (Heart - Atrial Appendage [-]; Heart - Left Ventricle [-])<br>*TNRC6C-AS1* (Heart - Atrial Appendage [-]; Heart - Left Ventricle [-])
rs379149	T	*TMC6* (Heart - Atrial Appendage [-]; Heart - Left Ventricle [-]; Artery - Tibial [-])<br>*TNRC6C-AS1* (Heart - Atrial Appendage [-]; Heart - Left Ventricle [-]; Thyroid [-])<br>*TMC8* (Whole Blood [+])
rs383603	G	*TMC6* (Whole Blood [-])
rs452483	T	*TMC6* (Heart - Atrial Appendage [-]; Heart - Left Ventricle [-])<br>*TNRC6C-AS1* (Heart - Atrial Appendage [-]; Heart - Left Ventricle [-])<br>*TMC8* (Whole Blood [+])

Supplementary Table S12: eQTL results on the novel lead variant rs2748427 and neighboring genes based on the iMethyl database

Gene	$CD4^+$ T cells			Monocytes		
	Beta	SE(Beta)	*P*	Beta	SE(Beta)	*P*
TNRC6C	-0.0018	0.0120	0.882	0.0304	0.0226	0.182
TNRC6C-AS1	-0.0069	0.0128	0.590	-0.0071	0.0151	0.640
TMC6	0.0040	0.0093	0.669	0.0087	0.0102	0.393
TMC8	-0.0171	0.0124	0.170	-0.0245	0.0127	0.057
C17orf99		not expressed			not expressed	
SYNGR2	-0.0052	0.0175	0.769	-0.0083	0.0178	0.641
TK1	-0.0198	0.0243	0.417	-0.0385	0.0197	0.053
AFMID	**-0.0794**	**0.0275**	**0.005**	**-0.0381**	**0.0179**	**0.036**
BIRC5	0.0001	0.0522	0.999		not expressed	

Results listed in bold were nominally significant ($P < 0.05$).

Supplementary Table S13: Functional annotation for GWS variants in the novel locus spanning *SIX3* and *SIX2* genes

SNP	Gene	Transcript ID	Effect	SIFT	PolyPhen
rs11305873	*AC012354.1*	AC012354.1-201	Intron variant	-	-
rs895636	*AC012354.1*	AC012354.1-201	Non-coding transcript exon variant	-	-
rs4953155	*AC012354.1*	AC012354.1-201	Intron variant	-	-
rs5830819	*AC012354.1*	AC012354.1-201	Intron variant	-	-
rs10168523	*AC012354.1*	AC012354.1-201	Intron variant	-	-
rs12712928	*AC012354.1*	AC012354.1-201	Intron variant	-	-

Supplementary Table S14: eQTL results from the GTEx database for GWS variants in the novel locus spanning *TMC6* and *TMC8* genes

SNP	EA[a]	GTEx[b]
rs11305873	G	No significant eQTLs were found in all eQTL Tissues
rs895636	T	No significant eQTLs were found in all eQTL Tissues
rs4953155	T	No significant eQTLs were found in all eQTL Tissues
rs5830819	AC	No significant eQTLs were found in all eQTL Tissues
rs10168523	G	No significant eQTLs were found in all eQTL Tissues
rs12712928	C	No significant eQTLs were found in all eQTL Tissues

[a]Effect allele; [b]Significant eQTL results (gene name, tissue, and effect direction) were shown.

Supplementary Table S15: eQTL results on the novel lead variant rs10168523 and neighboring genes based on the iMethyl database

Gene	CD4^{+} T cells			Monocytes		
	Beta	SE(Beta)	*P*	Beta	SE(Beta)	*P*
SIX3-AS1	not expressed			not expressed		
SIX3	not expressed			not expressed		
SIX2	not expressed			not expressed		

Supplementary Table S16: KEGG pathways that were nominally significantly associated with HbA_{1c}

Pathway	#. Genes	*P*
**LINOLEIC ACID METABOLISM**	**34**	**9.1E-07**
INSULIN SIGNALING PATHWAY	120	0.001
NUCLEOTIDE SUGARS METABOLISM	15	0.003
CELL CYCLE	97	0.004
STREPTOMYCIN BIOSYNTHESIS	10	0.006
TETRACHLOROETHENE DEGRADATION	9	0.006
BISPHENOL A DEGRADATION	13	0.013
BLOOD GROUP GLYCOLIPID BIOSYNTHESISLACTOSERIES	10	0.016
CALCIUM SIGNALING PATHWAY	158	0.016
MATURITY ONSET DIABETES OF THE YOUNG	19	0.017
SULFUR METABOLISM	10	0.021
ASCORBATE AND ALDARATE METABOLISM	14	0.022
PURINE METABOLISM	140	0.032
TYPE II DIABETES MELLITUS	43	0.033
FOCAL ADHESION	191	0.037
GAP JUNCTION	78	0.041

Results listed in bold were significant after multiple testing corrections ($P < 0.05/168$)

Supplementary Table S17: Gene-based *P*-values for genes in the KEGG linoleic acid metabolism pathway

Gene	Chr	Start	End	#. SNPs	*P*
PLA2G2E	**1**	**20,246,800**	**20,250,110**	**3**	**0.015**
PLA2G2A	1	20,301,924	20,306,932	6	0.869
PLA2G5	1	20,386,157	20,418,394	21	0.215
PLA2G2D	1	20,439,143	20,446,059	5	0.583
PLA2G2F	**1**	**20,465,823**	**20,476,879**	**8**	**0.029**
CYP2J2	1	60,358,980	60,392,445	30	0.260
PLA2G4A	1	186,798,032	186,958,113	34	0.166
RDH14	2	18,735,989	18,741,959	2	0.097
PLA2G12A	4	110,631,145	110,651,242	4	0.746
CYP3A5	7	99,245,813	99,277,636	9	0.201
CYP3A7	7	99,302,660	99,332,853	13	0.150
CYP3A4	7	99,354,583	99,381,811	5	0.593
CYP3A43	7	99,425,636	99,463,727	2	0.208
AKR1B10	**7**	**134,212,344**	**134,226,166**	**5**	**0.004**
ALOX5	10	45,869,624	45,941,569	23	0.422
PLA2G12B	10	74,694,519	74,714,590	1	0.188
CYP2C18	**10**	**96,443,251**	**96,495,947**	**19**	**0.028**
CYP2C19	**10**	**96,522,463**	**96,612,671**	**27**	**0.024**
CYP2C9	10	96,698,350	96,749,486	29	0.131
CYP2C8	**10**	**96,796,529**	**96,829,255**	**32**	**0.017**
CYP2E1	**10**	**135,340,300**	**135,352,627**	**24**	**0.027**
HSD17B12	11	43,702,108	43,878,169	24	0.218
FADS1	**11**	**61,567,097**	**61,584,529**	**4**	**0.003**
FADS2	**11**	**61,583,675**	**61,634,826**	**10**	**0.002**
FADS3	**11**	**61,640,998**	**61,659,006**	**3**	**0.005**
PLA2G1B	12	120,759,914	120,765,592	3	0.511

Results listed in bold were nominally significant genes ($P < 0.05$).

Supplementary Table S17: Gene-based *P*-values for genes in the KEGG linoleic acid metabolism pathway (cont.)

Gene	Chr	Start	End	#. SNPs	*P*
RDH11	14	68,143,517	68,162,510	2	0.337
RDH12	14	68,168,603	68,201,168	8	0.584
CYP1A2	**15**	**75,041,184**	**75,048,941**	**4**	**0.008**
HSD3B7	**16**	**30,995,949**	**31,000,473**	**1**	**0.028**
ALOX15	17	4,534,214	4,545,583	4	0.150
RDH13	19	55,555,684	55,580,914	13	0.075
PLA2G3	22	31,530,793	31,536,469	7	0.787
PLA2G6	22	38,507,502	38,577,857	19	0.108

Supplementary Table S18: Ingenuity pathways that were nominally significantly associated with HbA_{1c}

Pathway	#. Genes	*P*
**14-3-3-mediated Signaling**	**23**	**6.6E-05**
ERK MAPK Signaling	23	0.020
Antigen Presentation Pathway	11	0.024
Insulin Receptor Signaling	33	0.025
Xenobiotic Metabolism Signaling	37	0.034
RAR Activation	44	0.035
Cell Cycle G1 S Checkpoint Regulation	16	0.035
Aryl Hydrocarbon Receptor Signaling	46	0.039
Chemokine Signaling	21	0.045

Results listed in bold were significant after multiple testing corrections ($P < 0.05/92$)

Supplementary Table S19: Gene-based *P*-values for genes in the Ingenuity 14-3-3-mediated signaling pathway

Gene	Chr	Start	End	#. SNPs	*P*
TP73	1	3,569,129	3,652,765	33	0.089
RPS6KA1	1	26,856,249	26,901,520	13	0.374
JUN	1	59,246,463	59,249,785	1	0.219
RAF1	3	12,625,100	12,705,700	11	0.085
PDCD6IP	**3**	**33,840,063**	**33,911,199**	**10**	**0.025**
SNCA	4	90,645,250	90,759,447	15	0.347
TNF	6	31,543,344	31,546,113	2	0.932
MAP3K5	6	136,878,184	137,113,656	44	0.085
TRAF2	9	139,776,341	139,821,853	9	0.164
VIM	**10**	**17,269,934**	**17,279,592**	**2**	**0.045**
BAD	**11**	**64,037,300**	**64,052,176**	**7**	**0.043**
YAP1	11	101,981,151	102,104,154	21	0.588
CBL	11	119,076,986	119,178,859	12	0.202
TNFRSF1A	12	6,437,923	6,451,283	6	0.471
CDKN1B	**12**	**12,870,302**	**12,875,305**	**3**	**0.008**
FOXO1	13	41,129,801	41,240,734	17	0.052
FOS	14	75,745,477	75,748,937	2	0.241
GFAP	17	42,982,994	42,992,920	5	0.102
MAPT	17	43,971,702	44,105,700	15	0.206
GRB2	17	73,314,157	73,401,790	8	0.099
BAX	19	49,458,117	49,465,055	3	0.997
AKT1S1	19	50,372,290	50,381,613	7	0.336
SRC	20	35,973,088	36,033,835	10	0.077

Results listed in bold were nominally significant genes ($P < 0.05$).

Supplementary Table S20: PANTHER pathways that were nominally significantly associated with HbA_{1c}

Pathway	#. Genes	*P*
Pentose phosphate pathway	12	6.2E-04
S-adenosylmethionine biosynthesis	2	0.003
Insulin/IGF pathway-protein kinase B signaling cascade	32	0.014
FAS signaling pathway	21	0.016
Androgen/estrogene/progesterone biosynthesis	14	0.018
Integrin signalling pathway	119	0.021
Xanthine and guanine salvage pathway	4	0.050

No pathway was significantly associated after multiple testing correction ($P < 0.05/140$)

Supplementary Table S21: PANTHER molecular function categories that were nominally significantly associated with HbA_{1c}

Gene category	#. Genes	*P*
Voltage-gated potassium channel	84	0.002
Other ion channel	39	0.004
Phosphatase	6	0.004
Oxygenase	87	0.011
Carbohydrate kinase	23	0.013
Calmodulin related protein	122	0.023
Other viral protein	1	0.025
Adenylate cyclase	9	0.030
Non-receptor tyrosine protein kinase	33	0.030
Cysteine protease	88	0.048
Decarboxylase	19	0.049

No gene category was significantly associated after multiple testing correction ($P < 0.05/252$)

Supplementary Table S22: PANTHER biological process categories that were nominally significantly associated with HbA_{1c}

Gene category	#. Genes	*P*
Cation transport	411	0.002
DNA replication	86	0.004
Electron transport	129	0.007
Cell cycle control	320	0.008
Sex determination	8	0.013
Intracellular signaling cascade	213	0.021
Steroid metabolism	50	0.030
Other mRNA transcription	17	0.030
mRNA transcription termination	7	0.031
Cell communication	268	0.034
mRNA transcription initiation	49	0.034
Nitrogen utilization	1	0.037
Mitochondrial transport	23	0.037
Purine metabolism	55	0.038
Carbohydrate transport	45	0.040
Cell structure and motility	159	0.045
Protein methylation	10	0.045
Glycogen metabolism	34	0.048
Sulfur redox metabolism	19	0.049

No gene category was significantly associated after multiple testing correction ($P < 0.05/241$)

Supplementary Table S23: Top 20 GO term categories that were nominally significantly associated with HbA_{1c}

GO term	#. Genes	*P*
definitive erythrocyte differentiation	1	4.3E-05
response to follicle-stimulating hormone stimulus	1	4.3E-05
response to luteinizing hormone stimulus	1	4.3E-05
transforming growth factor beta receptor activity, type III	1	4.3E-05
magnesium ion binding	144	7.4E-05
regulation of establishment of protein localization	2	8.3E-06
nuclear origin of replication recognition complex	4	1.2E-04
GBD domain binding	1	1.3E-04
negative regulation of establishment of protein localization in plasma membrane	1	1.3E-04
histamine secretion by mast cell	2	2.1E-04
aromatase activity	21	2.5E-04
positive regulation of embryonic development	1	2.8E-04
cardiac epithelial to mesenchymal transition	3	3.5E-04
regulation of vesicle-mediated transport	1	3.7E-04
profilin binding	5	4.2E-04
nuclear replication fork	1	4.3E-04
regulation of protein stability	10	5.2E-04
alpha2-beta1 integrin complex	1	6.5E-04
positive regulation of glial cell proliferation	1	6.5E-04
positive regulation of stress-activated protein kinase signaling pathway	1	6.5E-04

No GO category was significantly associated after multiple testing correction ($P < 0.05/9,027$)

Supplementary Table S24: Association of the KEGG linoleic acid metabolism pathway with glycemic and erythrocyte-related traits

Trait	*P*
FPG	5.6E-01
GA	1.5E-01
GG	3.6E-01
eGFRcrea[a]	8.3E-01
eGFRcys[a]	5.1E-01
RBC	2.1E-01
**Hb**	**4.5E-02**
HCT	8.9E-02
MCV	3.8E-01
MCH	7.8E-01
MCHC	6.9E-01

Association between the KEGG linoleic acid metabolism and glycemic and erythrocyte-related traits were tested by combining variant-level evidence of association into pathway-level evidence. Pathway analyses was based on the Iwate population (n = 3,664). For FPG analysis, data was available for 604 subjects (n = 604). Results listed in bold were nominally significant ($P < 0.05$).

[a]log-transformed;

FPG, fasting plasma glucose; GA, glycated albumin; GG, glycation gap; eGFRcrea, estimated glomerular filtration rate based on creatinine; cGFRcys, estimated glomerular filtration rate based on cystatin; RBC, red blood cell count; Hb, hemoglobin concentration; HCT, hematocrit value; MCV, mean corpuscular volume; MCH, mean corpuscular hemoglobin; MCHC, mean corpuscular hemoglobin concentration

Supplementary Table S25: Association of the Ingenuity 14-3-3-mediated signaling pathway with glycemic and erythrocyte-related traits

Trait	*P*
FPG	5.9E-01
GA	5.7E-01
GG	3.1E-01
eGFRcrea[a]	6.6E-01
eGFRcys[a]	6.8E-01
RBC	8.3E-01
Hb	7.5E-01
HCT	7.4E-01
MCV	5.5E-02
MCH	4.2E-01
MCHC	8.9E-01

Association between the KEGG linoleic acid metabolism and glycemic and erythrocyte-related traits were tested by combining variant-level evidence of association into pathway-level evidence. Pathway analyses was based on the Iwate population (*n* = 3,664). For FPG analysis, data was available for 604 subjects (*n* = 604).

[a]log-transformed;

FPG, fasting plasma glucose; GA, glycated albumin; GG, glycation gap; eGFRcrea, estimated glomerular filtration rate based on creatinine; cGFRcys, estimated glomerular filtration rate based on cystatin; RBC, red blood cell count; Hb, hemoglobin concentration; HCT, hematocrit value; MCV, mean corpuscular volume; MCH, mean corpuscular hemoglobin; MCHC, mean corpuscular hemoglobin concentration
